# Draft genome of the Marco Polo Sheep (*Ovis ammon polii*)

**DOI:** 10.1093/gigascience/gix106

**Published:** 2017-11-01

**Authors:** Yongzhi Yang, Yutao Wang, Yue Zhao, Xiuying Zhang, Ran Li, Lei Chen, Guojie Zhang, Yu Jiang, Qiang Qiu, Wen Wang, Hong-Jiang Wei, Kun Wang

**Affiliations:** Center for Ecological and Environmental Sciences, Northwestern Polytechnical University, Xi'an 710072, China; College of Life and Geographic Sciences, Kashgar University, Kashgar 844000, China; The Key Laboratory of Ecology and Biological Resources in Yark and Oasis at Colleges and Universities under the Department of Education of Xinjiang Uygur Autonomous Region, Kashgar University, Kashgar 844000, China; College of Animal Science and Technology, Northwest A&F University, Yangling 712100, China; Centre for Social Evolution, Department of Biology, Universitetsparken 15, University of Copenhagen, Copenhagen 2100, Denmark; Key Laboratory Animal Nutrition and Feed of Yunnan Province, Yunnan Agricultural University, Kunming 650201, China

**Keywords:** annotation, evolution, genome assembly, Marco Polo Sheep

## Abstract

**Background:**

The Marco Polo Sheep (*Ovis ammon polii*), a subspecies of argali (*Ovis ammon*) that is distributed mainly in the Pamir Mountains, provides a mammalian model to study high altitude adaptation mechanisms. Due to over-hunting and subsistence poaching, as well as competition with livestock and habitat loss, *O. ammon* has been categorized as an endangered species on several lists. It can have fertile offspring with sheep. Hence, a high-quality reference genome of the Marco Polo Sheep will be very helpful in conservation genetics and even in exploiting useful genes in sheep breeding.

**Findings:**

A total of 1022.43 Gb of raw reads resulting from whole-genome sequencing of a Marco Polo Sheep were generated using an Illumina HiSeq2000 platform. The final genome assembly (2.71 Gb) has an N50 contig size of 30.7 Kb and a scaffold N50 of 5.49 Mb. The repeat sequences identified account for 46.72% of the genome, and 20 336 protein-coding genes were predicted from the masked genome. Phylogenetic analysis indicated a close relationship between Marco Polo Sheep and the domesticated sheep, and the time of their divergence was approximately 2.36 million years ago. We identified 271 expanded gene families and 168 putative positively selected genes in the Marco Polo Sheep lineage.

**Conclusions:**

We provide the first genome sequence and gene annotation for the Marco Polo Sheep. The availability of these resources will be of value in the future conservation of this endangered large mammal, for research into high altitude adaptation mechanisms, for reconstructing the evolutionary history of the *Caprinae*, and for the future conservation of the Marco Polo Sheep.

## Data Description

### Introduction to *O. ammon polii*

The Marco Polo Sheep (*Ovis ammon polii*) is a subspecies of argali (*Ovis ammon*), named after the explorer Marco Polo. It was first described scientifically in 1841 by Edward Blyth [1]. This subspecies is distributed mainly in the Pamir Mountains, which consist of rugged ranges at elevations of 3500–5200 meters [2]. The habitat of the subspecies includes the Tajikistan Pamir Mountains [3], as well as limited regions in China, Afghanistan, Pakistan, and Kyrgyzstan [4]. The Marco Polo Sheep species represents a new model to study high altitude adaptation mechanisms adopted by mammals. Due to the sheep's impressively long horns, foreign hunters have for many years been willing to pay large amounts of money to take part in a hunt [5], and this is still the case today [2]. Recent studies on the status of the argali population have shown a decline in numbers, caused mainly by over-hunting and subsistence poaching, as well as by competition with livestock and habitat loss [6–9]. *O. ammon* has been categorized in several protection lists, such as Appendix II of the Convention on International Trade in Endangered Species of Wild Fauna and Flora (CITES) and the International Union for Conservation of Nature and Natural Resources (IUCN) Red List, as a vulnerable or nearly threatened species. Conservation and restoration measures are therefore needed in order to safeguard the species, and information about its genome will be a key element in formulating an appropriate conservation strategy.

### Sequencing

High–molecular weight genomic DNA was extracted from fibroblast cells cultured from the ear skin biopsy sample of a male *O. ammon polii* using a Qiagen DNA purification kit. The sheep was originally captured from the Pamir Plateau of China and reared in the KaShi Zoo, Kashgar Prefecture, Xinjiang Province, China. A whole-genome shotgun sequencing strategy was applied, and a series of libraries with insert seizes ranging from 400 base pairs (bp) to 15 kilobase pairs (kb) were constructed using the standard protocol provided by Illumina (San Diego, CA, USA). To construct small-insert libraries (400, 500, 600, 700, and 800 bp), DNA was sheared to the target size range using a Covaris S2 sonicator (Covaris, Woburn, MA, USA) and ligated to adaptors. For long-insert libraries (4, 8, 10, 12, and 15 kb), DNA was fragmented using a Hydroshear system (Digilab, Marlborough, MA, USA). Sheared fragments were end-labeled with biotin, and fragments of the desired size were gel purified. A second round of fragmentation was then conducted before adapter ligation. All libraries were sequenced on an Illumina HiSeq 2000 platform (Table S1). A total of 1022.43 Gb of raw data was generated, and 624.74 Gb of clean data was retrieved after removal of duplicates, contaminated reads (reads with adaptor sequence), and low-quality reads using the sickle software tool [10] with a quality threshold of 10 and a length threshold of 50. We further corrected the short-insert library reads using SOAPec [11], a k-mer-based error correction package.

### Evaluation of genome size

Approximately 65 Gb of clean reads were randomly selected from all short libraries to estimate the genome size using the k-mer-based method and the formula G = k-mer_number/k-mer_depth. In this study, a total of 52 413 427 492 k-mers were generated and the peak k-mer depth was 17. The genome size was estimated to be approximately 3 Gb (Table S2 and Fig. S1), and all the clean data correspond to a coverage of ∼208-fold.

### *De novo* genome assembly

The assembly was performed using Platanus v1.2.4 (Platanus, RRID:SCR_015531) [12], which is well suited to high-throughput short reads and heterozygous diploid genomes. Briefly, error-corrected paired-end reads (insert size < 2 kb) were input for contig assembly with the default parameters. Next, all cleaned paired-end (insert size < 2 kb) reads and mate-paired (insert size > 2 kb) reads were mapped onto the contigs for scaffold building using default parameters, except that the minimum number of links (–l) was set to 10 in order to minimize the number of scaffolding errors. After gap filling by Platanus, the gaps that still remained in the resulting scaffolds were closed using GapCloser (GapCloser, RRID:SCR_015026) [11]. The final *de novo* assembly for the Marco Polo Sheep has a total length of 2.71 Gb, including 116.91 Mb (4.3%) of unknown bases. The assembly is slightly larger than that of the domestic sheep (*Ovis aries*, Oar_v3.1, 2.61 Gb) [13] and smaller than that of the domestic goat (*Capra hircus*, ARS1, 2.92 Gb) [14]. The N50s for contigs and scaffolds of the Marco Polo Sheep genome are, respectively, 30.8 kb and 5.5 Mb (Table S3). The assembled scaffolds represented ∼ 88% of the estimated genome size, and the GC content was 41.9%, similar to those of sheep (41.9%) and goat (41.5%) (Fig. S2).

We assessed the quality of the genome assembly with respect to base-level accuracy, integrity, and continuity. More than 99.65% of the short-insert paired-end reads could be mapped to the assembly, and more than 98.35% of the sequence has a coverage depth greater than 20-fold (Table S4); thus the assembly is of high-level single-base accuracy. A core eukaryotic genes (CEG) mapping approach (CEGMA, RRID:SCR_015055, v2.5) [15] dataset comprising 248 CEGs was used to evaluate the completeness of the draft: 93.55% (232/248) of genes were completely or partially covered in the assembled genome (Table S5). Alongside this, we also used the Benchmarking Universal Single-Copy Orthologs (BUSCO, RRID:SGR_015008, v2.0.1 [16]: the representative mammal gene set mammalia_odb9, which contains 4104 single-copy genes that are highly conserved in mammals) software package to assess the quality of the genome assembly generated. The resulting BUSCO value was 95.9% (containing C = 92.5%, S = 91.3%, D = 1.2%, F = 3.4%, M = 4.1%, n = 4104; C: complete, D: duplicated, F: fragmented, M: missed, n: genes) (Table S6). Both the CEGMA and the BUSCO scores are comparable with those for the sheep (Oar_v3.1) and domestic goat (ARS1 and CHIR_1.0), which are known for their high quality of the reference genomes of 2 important livestock animals, suggesting our Marco Polo Sheep assembly is of high quality and quite complete. Finally, to evaluate the trade-off between the contiguity and correctness of our assembly, we applied the feature-response curve (FRC) method [17], which predicts the correctness of an assembly by identifying “features” representing potential errors or complications on each *de novo* assembled scaffold during the assembly process. The FRC curve was calculated for the Marco Polo Sheep, sheep, taurine cattle, and 2 versions of goat assemblies (Fig. S3). We found that the curve for our assembly was similar to that for the sheep and the 2 goat assemblies, with taurine cattle slightly different from the others, indicating that the level of contiguity and correctness of the Marco Polo Sheep genome assembly is comparable to those of sheep and goat.

We mapped the reads from short-insert length libraries to the Marco Polo Sheep reference genome with BWA (BWA, RRID:SCR_010910) [18] and performed variant calling with SAMtools v0.1.19 (SAMTOOLS, RRID:SCR_002105) [19]. Applying strict quality control and filtering, we obtained a total of 3.5 million SNVs (Table S7) and noted that the heterozygosity rate (0.14%) was lower than that estimated for sheep (Oar_v3.1, 0.2%) and similar with that of goat (CHIR_1.0, 0.13%) [13]. We further assessed the distribution of heterozygosity ratio of non-overlapping 50K windows (Fig. S4). We assume that the heterozygosity on the genome can be divided into 3 states (low/normal/high) and applied the Hidden Markov model with the depmixS4 package [20] in R to infer the state of each window. The genomic regions with “low heterozygosity” state that 14% of the genome was highly homozygous (mean heterozygosity rate = 0.003%), which could be explained by either loss of polymorphism in endangered species [21] or recent inbreeding in some specific Marco Polo sheep individuals. More samples will be required to test whether the highly homozygous status was common in this species. One hundred fifty-six genes overlapped with more than half of the length in the low-heterozygosity regions, and the GO enrichment analysis shows that no GO category was significantly enriched (Table S8). A total of 384 018 insertions and deletions (InDels) (Table S9) were obtained. Similar to the findings of previous studies on yak [22] and wisent [23], the InDels in the coding regions were enriched for sizes that are multiples of 3 bases (Fig. S5).

### Annotation

The transposable elements present in Marco Polo sheep sequences were identified using a combination of *de novo–* and homology-based approaches. Transposable elements were identified at both the DNA and the protein levels, based on known sequences contained within the DNA repeat database (RepBase v21.01) [24], using RepeatMasker v4.0.5 (RepeatMasker, RRID:SCR_012954) [25] and RepeatProteinMask (v4.0.5, a package within RepeatMasker). For the *de novo* prediction, first RepeatModeler V1.0.8 (RepeatModeler, RRID:SCR_015027) was employed to construct a *de novo* repeat library, and then RepeatMasker was used to identify repeats using both the *de novo* repeat database and RepBase. We then combined the *de novo* prediction and the homolog prediction of transposable elements according to the coordination in the genome. Tandem repeats were annotated with RepeatMasker and Tandem Repeats Finder (TRF; v4.07) [26]. In summary, a total of 0.87% tandem repeats and 46.60% transposable elements were identified in the Marco Polo sheep assembly, with LINEs constituting the greatest proportion, 72.48% of all repeats, and SINEs making up 24.09% of all repeats (Table S10 and Table S11).

We used homology-based and *de novo* prediction to annotate protein coding genes. For homology-based prediction, protein sequences from 5 different species (*Bos taurus*, *Equus caballus*, *Homo sapiens*, *Ovis aries*, *Sus scrofa*) (Table S12) were mapped onto the repeat-masked Marco Polo sheep genome using TblastN with an E-value cutoff of 1e-5; the aligned sequences as well as the corresponding query proteins were then filtered and passed to GeneWise (GeneWise, RRID:SCR_015054) [27] to search for accurately spliced alignments. For *de novo* prediction, we first randomly selected 1500 full-length genes from the results of homology-based prediction to train the model parameters for Augustus v3.2.1 (Augustus: Gene Prediction, RRID:SCR_008417) [28] and geneid v1.4.4 [29]. GenScan [30], Augustus v3.2.1 [28], and geneid v1.4.4 [29] were then used to predict genes based on the training set of human and Marco Polo Sheep genes. We used EVidenceModeler software (EVM; v1.1.1) to integrate the genes predicted by the homology and *de novo* approaches and generated a consensus gene set (Table S13). The final gene set was produced by removing low-quality genes of short length (proteins with fewer than 50 amino acids) and/or exhibiting premature termination. The final total gene set consisted of 20 336 genes, and the number of genes, gene length distribution, and exon number per gene were similar to those of other mammals, while the intron length was slightly larger than goat (CHIR_1.0), sheep (Oar_v3.1), and taurine cattle (UMD3.1) (Table S14 and Figs S6, S7). The repeat content was annotated by RepeatMasker v4.0.5 [25] with unified parameters for Marco Polo sheep, domestic sheep, and goat. We found that there were more LINE sequences in the intron regions of Marco Polo sheep than the other species, suggesting that transposon insertions might have contributed to intron length increasing (Fig. S8); 92.55% of all the predicted genes could be annotated using 5 protein databases: InterPro (InterPro, RRID:SCR_006695) (87.17%), gene ontology (GO; 70.99%), Swiss-Prot (91.67%), TrEMBL (92.33%), and Kyoto Encyclopedia of Genes and Genomes (KEGG, RRID:SCR_012773; 57.25%) (Table S15). In addition, we identified 2978 non-coding RNAs in the Marco Polo Sheep genome (Table S16).

### Genome evolution

First, large-scale variations among Marco Polo Sheep, sheep, and goat were identified by synteny analysis using the program LAST (LAST, RRID:SCR_006119) [31]. A total of 2.29/2.30/2.40 Gb 1:1 alignment sequences were generated for, respectively, Marco Polo Sheep vs sheep (Oar_v3.1), Marco Polo Sheep vs goat (ASR1), and sheep vs goat, covering more than 88.55% of each genome (Table S17 and Fig. S9). The sequences present on sheep/goat autosomes were well covered (average values: 89.65%/89.88%) by the synteny alignment, whereas only 66.09%/63.03% were covered in the case of chromosome X. The scaffolds of the Marco Polo Sheep genome that aligned to the sex chromosomes were also more fragmented. The divergence rates between Marco Polo Sheep vs sheep (Oar_v3.1), Marco Polo Sheep vs goat (ASR1), and sheep vs goat were 0.7%, 2.2%, and 2.3%, respectively, corresponding to their relatedness (Table S17 and Fig. S10). Although Marco Polo Sheep, sheep, and goat showed good synteny alignments, there are large numbers of inter-chromosomal rearrangements between pairs of them (Figs S11 and S12). By comparing Marco Polo Sheep and sheep/goat genomes, we identified 11 756/6026 inter-chromosomal, intra-chromosomal, or inversion breakpoints (edges of transposition events) (Table S18), which may have been caused by the real translocations events between them as they have a different karyotype, errors in the assembly of the genomes, or erroneous synteny alignments (false positives and false negatives). However, at this stage it is difficult to distinguish between possible artifactual and real effects. The breakpoint distributions were significantly enriched in repeat regions (Fig S13a), which are susceptible to rearrangements but also to assembly or alignment errors. Longer scaffolds were found to harbor fewer breakpoints (Fig. S13b). Single molecule sequencing with unbiased long reads will be the best way of identifying large-scale variation.

**Figure 1:**
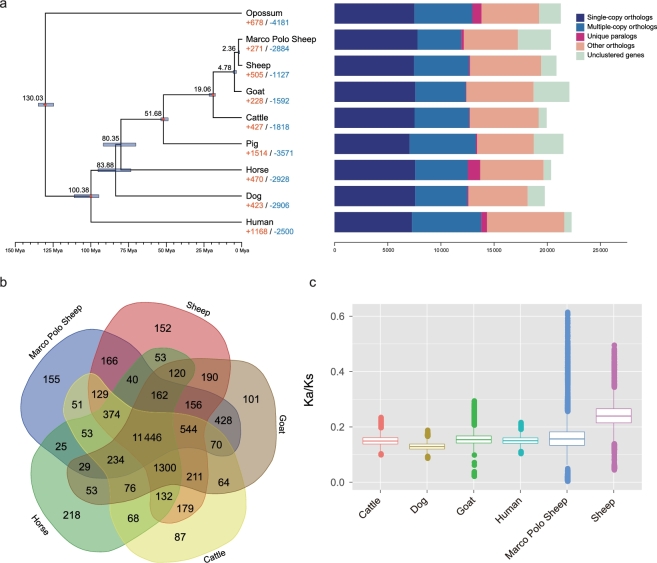
Phylogenetic relationships and genomic comparisons between Marco Polo Sheep and other mammals. (**a**) Divergence time estimates for the 9 mammals generated using MCMCtree and the 4-fold degenerate sites. The red dots correspond to calibration points and the divergence times. Divergence time estimates (Mya) are indicated above the appropriate nodes; blue nodal bars indicate 95% confidence intervals. Gene orthology was determined by comparing the genomes with the OrthoMCL software. (**b**) A Venn diagram of the shared orthologues among Marco Polo Sheep, sheep, goat, taurine cattle, and horse. Each number represents a gene family number. (**c**) The box plot shows the ratio of non-synonymous to synonymous mutations (Ka/Ks) for Marco Polo Sheep, sheep, goat, taurine cattle, horse, and human.

To analyze gene families, we downloaded the protein sequences of 8 additional species (Opossum, human, dog, horse, pig, taurine cattle, goat, and sheep) from Ensembl (Ensembl, RRID:SCR_002344) [32] and *Giga*DB (*Giga*DB, RRID:SCR_004002) (Table S12) [33]. The consensus gene set for the above 8 species and Marco Polo Sheep were filtered to retain the longest coding sequence (CDS) for each gene, removing CDS with premature stop codons and those protein sequences <50 amino acids in length, resulting in a dataset of 188 359 protein sequences, which was used as the input file for OrthoMCL (OrthoMCL DB: Ortholog Groups of Protein Sequences, RRID:SCR_007839) [34]. A total of 17 578 OrthoMCL families were built utilizing an effective database size of all-to-all BLASTP strategy with an E-value of 1e-5 and a Markov Chain Clustering default inflation parameter (Table S19 and Fig. 1a). We identified 155 gene families that were specific to the Marco Polo Sheep when comparing with taurine cattle, sheep, goat, and horse (Fig. 1b) and detected 271 gene families that have expanded in the Marco Polo Sheep lineage using Computational Analysis of gene Family Evolution, v4.0.1 (CAFÉ) (Fig. 1a) [35]. The expanded gene families were enriched in 38 GO categories, and their functions were mainly associated with response to stimulus, cell adhesion, G-protein coupled receptor, and enzyme activity (Table S20).

Next, we selected 5788 single-copy gene families from the above-mentioned 9 mammalian species and used PRANK v3.8.31 [36] with the codon option to align the CDS from each single-copy gene family. Four-fold degenerate sites were extracted from all the single-copy genes and used to construct a phylogenetic tree with the GTR+G+I model in RAxML v7.2.8 (RAxML, RRID:SCR_006086) (Fig. S14) [37]. The divergence time of each node was estimated by the PAML (PAML, RRID:SCR_014932) MCMCtree program v4.5 [38] and calibrated against the timing of the divergence of the opossum and human (124.6–134.8 million years ago [Mya]), human and taurine cattle (95.3–113 Mya), taurine cattle and pig (48.3–53.5 Mya), and taurine cattle and goat (18.3–28.5 Mya) [39]. The convergence was checked by Tracer v1.5 [40] and confirmed by 2 independent runs. The phylogenetic analysis showed that the Marco Polo Sheep has a closer relationship with sheep than with other mammals and that the divergence time between them is about 2.36 (1.94–2.61) Mya (Fig. 1a).

We further used the free ratio model to calculate the average Ka/Ks values and the branch-site likelihood ratio test to identify positively selected genes in the Marco Polo Sheep lineage. A total of 10 353 high-confidence single-copy genes were identified by InParanoid and MultiParanoid within the human, dog, taurine cattle, goat, sheep, and Marco Polo Sheep. We found that the Marco Polo Sheep has a regular level of the average Ka/Ks values, but containing more outliers (Fig. 1c). A total of 168 positively selected genes were identified in the Marco Polo Sheep lineage (Table S21), and 6 of them were orthologous with high altitude adaptation-related genes (IDE, IGF1, P2RX3, PHF6, PROX1, and RYR1) identified in Tibet wild boar [41]. Two genes were associated with hypoxia response: the ryanodine receptor protein encoded by ryanodine receptor 1 (*RYR1*) was located in the pulmonary artery smooth muscle cells, which could subserve coupled O_2_ sensor and NO regulatory functions to respond to the tissue hypoxic decrease [42]; purinergic receptor P2X, ligand-gated ion channel 3 (*P2RX3*) is reported as a potential new target for the control of human hypertension, which could reduce the arterial pressure and basal sympathetic activity and normalize carotid body hyperreflexia in conscious rats with hypertension during P2RX3 antagonism [43]. Four genes were related with energetic metabolism: insulin-like growth factor 1 (*IGF1*) encodes the growth-promoting polypeptide mainly involved in body growth and differentiation as well as the glucose, lipid, and protein metabolism [44]; insulin degrading enzyme (*IDE*) encodes a zinc metallopeptidase that degrades intracellular insulin, which could accelerate glycolysis, pentose phosphate cycle, and glycogen synthesis in the liver [45]; PHD finger protein 6 (*PHF6*) encodes a protein with 2 PHD-type zinc finger domains, and its function was associated with Börjeson-Forssman-Lehmann syndrome, which is 1 of the syndromic obesities in humans [46]; the protein encoded by prospero homeobox 1 (*PROX1*) could occupy promoters of metabolic genes on a genome-wide scale to control energy homeostasis [47]. In addition, the other identified PSGs may also be associated to high altitude adaptation, while literature data on their function are rare. Further studies will be required to clarify the roles of these genes in high altitude tolerance.

Finally, we inferred the demographic history of the Marco Polo Sheep using the Pairwise Sequentially Markovian Coalescent (PSMC) model [48]. Consensus sequences were obtained using SAMtools v0.1.19 [19] and divided into non-overlapping 100-bp bins. The analysis was performed with the following parameters: -N25 -t15 -r5 -p “4+25 × 2+4+6.” PSMC modeling was done using a bootstrapping approach, with sampling performed 100 times to estimate the variance of the simulated results. The effective population size (*N_e_*) of Marco Polo Sheep shows a peak at ∼1 Mya, followed by 2 distinct declines. The most recent decline involved at least a 7-fold decrease in *N_e_*, and it occurred ∼60 000 years ago (Fig. S15).

## Conclusion

In summary, the novel genome data generated in this work will provide a valuable resource for studying high altitude adaptation mechanisms within mammals and for investigating the evolutionary histories of the *Caprinae*, and they will have relevance for the future conservation of the Marco Polo Sheep.

## Availability of supporting data

The sequencing reads of each sequencing library have been deposited at NCBI with the Project ID PRJNA391748, Sample ID SAMN07274464, and the Genome Sequence Archive [49] in BIG Data Center, Beijing Institute Genomics (BIG), Chinese Academy of Science, under accession number PRJCA000449 (publicly accessible at http://bigd.big.ac.cn/gsa). The assembly and annotation of the Marco Polo Sheep genome are available in the *GigaScience* database, *Giga*DB (*Giga*DB, RRID:SCR_004002) [50]. Supplementary figures and tables are provided in Additional file 1.

## Additional Files

Figure S1. 21-mer-based analysis carried out to estimate the size of the Marco Polo Sheep genome.

Figure S2. GC content distribution for the genomes of Marco Polo Sheep, goat, and sheep.

Figure S3. FRCurve of 5 genome assemblies.

Figure S4. The distribution of observed heterozgosity stats within Marco Polo Sheep genome.

Figure S5. Counts of InDels in coding regions, showing an enrichment of multiples of 3 bases.

Figure S6. Comparison of gene structure characteristics with those of other mammals.

Figure S7. Comparison of gene structure characteristics of the 1:1 orthologs in the 5 mammals.

Figure S8. Comparison of the repeat content in the intron regions among Marco Polo Sheep, sheep (Oar_v3.1), and goat (CHIR_1.0).

Figure S9. Summary of the number of chromosomes to which a given scaffold of the Marco Polo Sheep genome could be aligned.

Figure S10. Divergence between Marco Polo Sheep, sheep, and goat.

Figure S11. Synteny relationship between Marco Polo Sheep and sheep.

Figure S12. Synteny relationship between Marco Polo Sheep and goat.

Figure S13. Density of breakpoints (number per million bases) in different regions of the genome.

Figure S14. Phylogeny relationships between Marco Polo Sheep and other mammals.

Figure S15. Demographic history of Marco Polo Sheep.

Table S1. Summary of sequenced reads.

Table S2. Estimation of genome size based on 21-mer statistics.

Table S3. Statistics for the final assemblies of the Marco Polo Sheep genome.

Table S4. Numbers of reads mapped to the assembled Marco Polo Sheep genome.

Table S5. Summary of CEGMA analysis results.

Table S6. Summary of BUSCO analysis results obtained by counting matches to 4104 single-copy orthologs (mammalia_odb9).

Table S7. The distribution of SNVs in the Marco Polo Sheep genome.

Table S8. Genes located in the low-heterozygosity regions.

Table S9. The distribution of InDels in the Marco Polo Sheep genome.

Table S10. Prediction of repetitive elements in the assembled Marco Polo Sheep genome.

Table S11. Classification of interspersed repeats in the assembled Marco Polo Sheep genome.

Table S12. Data on all species used during the genome analysis.

Table S13. Prediction of protein-coding genes in the Marco Polo Sheep.

Table S14. Comparative gene statistics.

Table S15. Functional annotation of predicted genes in the Marco Polo Sheep.

Table S16. Summary statistics of non-coding RNAs in the Marco Polo Sheep.

Table S17. Summary of synteny alignments.

Table S18. Summary of breakpoints between Marco Polo Sheep, sheep, and goat.

Table S19. Summary statistics of gene families in 9 species.

Table S20. Candidate PSGs in the Marco Polo Sheep lineage.

## Abbreviations

BLAST: basic local alignment search tools; bp: base pair; BUSCO: Benchmarking Universal Single-Copy Orthologs; CDS: coding sequence; CEGMA: a core eukaryotic genes mapping approach; CITES: the Convention on International Trade in Endangered Species of Wild Fauna and Flora; FRC: feature-response curve; InDels: insertion and deletions; Gb: giga bases; kb: kilo bases; GO: gene ontology; IUCN: the International Union for Conservation of Nature and Natural Resources; KEGG: Kyoto Encyclopedia of Genes and Genomes; LINE: long interspersed nuclear elements; Mb: mega bases; Mya: million years ago; Ne: effective population size; PSMC: pairwise sequentially Markovian coalescent; SINE: short interspersed nuclear element; SNV: single-nucleotide variants; SRA: Sequence Read Archive.

## Competing Interests

The authors declare that they have no competing interests.

## Author Contributions

K.W. and W.W. conceptualized the research project. K.W., W.W., and H.W. designed analytic strategy and coordinated the project. Y.W., H.W., and W.W. collected the samples and led the genome sequencing. Y.Y. and K.W. led the bioinformatics analysis. Y.Y., Y.W., and Y.Z. generated the genome assembly and the genome annotation. Y.Y., R.L., and L.C. finished the synteny analysis. Y.W., Y.Z., and G.Z. performed the gene family construction and the phylogeny analysis. Y.Y. and Q.Q. detected the PSGs and carried out data submission. Y.Y., W.W., and K.W. wrote the paper. All authors read and approved the final manuscript.

## Supplementary Material

GIGA-D-17-00160_Original-Submission.pdf

GIGA-D-17-00160_Revision-1.pdf

GIGA-D-17-00160_Revision-2.pdf

Response-to-Reviewer-Comments_Original-Submission.pdf

Response-to-Reviewer-Comments_Revision-1.pdf

Reviewer-1-Report-(Original-Submission).pdf

Reviewer-2-Report-(Original-Submission).pdf

Reviewer-2-Report-(Revision-1).pdf

Supplementary figures and tables
