# Supplementary material for: Draft genome of the Marco Polo Sheep (Ovis ammon polii)
Source: Gigascience. 2017 Nov 1;6(12):1–7. doi: 10.1093/gigascience/gix106 (PMC5740985; doi:10.1093/gigascience/gix106)
Supplement: GIGA-D-17-00160_Revision-1.pdf [file gix106_giga-d-17-00160_revision-1.pdf]

GigaScience

The genome of the Marco Polo Sheep (Ovis ammon polii)

--Manuscript Draft--

**Manuscript Number:**	GIGA-D-17-00160R1
**Full Title:**	The genome of the Marco Polo Sheep (Ovis ammon polii)
**Article Type:**	Data Note
**Funding Information:**	National Natural Science Foundation of China (31072019) — Dr. Yutao Wang<br>National Natural Science Foundation of China (31572381) — Dr. Yu Jiang<br>Talents Team Construction Fund of Northwestern Polytechnical University (NWPU) — Dr. Qiang Qiu, Dr. Wen Wang
**Abstract:**	Background: The Marco Polo Sheep (Ovis ammon polii), a subspecies of argali (Ovis ammon) which is distributed mainly in the Pamir Mountains, provides a mammalian model in which to study high-altitude adaptation mechanisms. Due to over-hunting and subsistence poaching, as well as competition with livestock and habitat loss, O. ammon has been categorized as an endangered species on several lists. It can have fertile offspring with sheep. Hence a high quality reference genome of the Marco Polo Sheep will be very helpful in conservation genetics and even in exploiting useful genes in sheep breeding.<br>Findings: A total of 1,022.43 Gb of raw reads resulting from whole-genome sequencing of a Marco Polo Sheep were generated using an Illumina HiSeq2000 platform. The final genome assembly (2.71 Gb), which has an N50 contig size of 30.7 Kb and a scaffold N50 of 5.49 Mb. The repeat sequences identified account for 46.72% of the genome and 20,336 protein-coding genes were predicted from the masked genome. Phylogenetic analysis indicated a close relationship between Marco Polo Sheep and the domesticated sheep, and the time of their divergence was approximately 2.36 million years ago (Mya). We identified 271 expanded gene families and 168 putative positively selected genes in the Marco Polo Sheep lineage.<br>Conclusions: We provide the first genome sequence and gene annotation for the Marco Polo Sheep. The availability of these resources will be of value in the future conservation of this endangered large mammal, for research into high-altitude adaptation mechanisms, for reconstructing the evolutionary history of the Caprinae and for the future conservation of the Marco Polo Sheep.
**Corresponding Author:**	Kun Wang, Ph. D<br>Northwestern Polytechnical University<br>Xi'an, Shannxi CHINA
**Corresponding Author Secondary Information:**	
**Corresponding Author's Institution:**	Northwestern Polytechnical University
**Corresponding Author's Secondary Institution:**	
**First Author:**	Yongzhi Yang
**First Author Secondary Information:**	
**Order of Authors:**	Yongzhi Yang<br>Yutao Wang<br>Yue Zhao<br>Xiuying Zhang<br>Ran Li

Powered by Editorial Manager® and ProduXion Manager® from Aries Systems Corporation

	Lei Chen
	Guojie Zhang
	Yu Jiang
	Qiang Qiu
	Wen Wang
	Hongjiang Wei
	Kun Wang, Ph. D
**Order of Authors Secondary Information:**	
**Response to Reviewers:**	Response to Referees<br>Your manuscript "The genome of the Marco Polo Sheep (Ovis ammon polii)" (GIGA-D-17-00160) has been assessed by our reviewers. Based on these reports, and my own assessment as Editor, I am pleased to inform you that it is potentially acceptable for publication in GigaScience, once you have carried out some essential revisions suggested by our reviewers.<br>Their reports are below.<br>The main points of the reviewers concern places in the manuscript where your wording should be more exact, or where you should be more careful with your interpretations (for example, the statement on effective population size, based on variation in just a single animal, may not be warranted, as pointed out by reviewer 1).<br>Reply: We are grateful for the editor and reviewers' helpful suggestions and instructions.<br>I note that you indicate several corresponding authors. OUP has a policy of only taking one - most responsive - author as corresponding author. The definition of corresponding authorship is one of responsiveness rather than seniority. The corresponding author is the one individual who takes primary responsibility for communication with the journal during the manuscript submission, peer review, and publication process. Please refer to the information below on our homepage and please decide based on these guidelines who should be corresponding author.<br>Reply: Nowadays most genome projects are results of collaborative results of several groups. In this work, all the three corresponding authors were made significant contributions in designing the project and organizing the manuscript, and thus we listed three correspondent authors with Dr. Kun Wang as the first correspondent author to communicate with the editorial office. We have listed the detail contributions in the section of Authors' contributions in the revised manuscript, which is highlighted with yellow.<br>You indicate several "co-first" authors with equal contributions. Please explain in more detail in how far the contributions of all three first authors are exactly equal. See also the CASRAI guidelines to define authorship roles:<br>http://dictionary.casrai.org/Contributor_Roles<br>Reply: All of three "co-first" authors were the major material collectors and data analysts, and have taken part in the paper's writing in this work. We have classified their works and listed the detail contribution of all authors in the section of Authors' contributions in the revised manuscript. Those three authors were evaluated and each of them should be listed as "co-first" authors.<br><br>Reviewer #1: The manuscript describes the construction of a draft assembly of the Marco Polo Sheep to an equivalent standard to the sheep, goat and cattle genomes. The assembly parameters are in line with what can reasonably be expected for the approach used. The text adequately documents the assembly and analysis methodologies. The analysis results are also consistent with expectations.<br>However, I think that the use of variation in a single animal to make such a strong statement about effective population size, and endangered population, of Marco Polo Sheep may not be warranted (lines 145-146).<br>Reply: We appreciate the reviewer's positive comments on this work. We agree that only one single sample could not represent the population size of a species. We have therefore removed the related statement, and furthermore we have added PSMC analysis to provide estimation on the demographic history, which is a highly reliable method to estimate demographic history using a single genome data.<br>I am also concerned about Table S21, where enrichment of potentially selected genes

Powered by Editorial Manager® and ProduXion Manager® from Aries Systems Corporation

in GO category is shown. The p-values shown are marginal for p-values adjusted for multiple testing (I generally disregard adjusted p-values of >10-3). However unlike other tables the p-values are not indicated as adjusted. If they are not adjusted the table should be omitted and the wording altered at lines 252/3. I note that "enriched" rather than "significantly enriched" is used in the text, but readers will tend to assume "significantly enriched" from such a mention in the text even if it is not stated that way.
Reply: Thanks for the suggestion, and we agree that the enrichment significance was marginal and thus we have replaced the GO category enrichment result with a paragraph to discuss some specific PSGs to make sure that all conclusions in the manuscript are solid.
Line 57, "long horns" rather than "long horn"
Reply: Corrected as suggested.
Lines 110/111, it is not clear exactly which versions of the sheep and goat genomes are being used here, one would have to check the references to find out, whereas elsewhere specific versions are mentioned in the text. I suggest that assembly version designations consistent with those used elsewhere are included here.
Reply: We apologize for causing this confusion and have added the information of the published genomes' versions that we used in our analysis in the revised manuscript.
Line 130 two goat assemblies are mentioned without references. How do they relate to each other and which assembly is being used on other occasions where only one goat genome assembly is mentioned? This is not clear in a number of the tables and at places in the text.
Reply: We have added the information of corresponding references, relationship and which version we have used in this revised manuscript.

Reviewer #2: This manuscript describes the genome sequencing and assembly of Marco Polo sheep (Ovis ammon poliii) a wild sheep species that is interesting in several regards. First, the species is adapted to living at high altitude. Second, the species is of conservation concern due to hunting, habitat loss and a limited range. Finally, there remains some uncertainty as to how often and where sheep were domesticated, and O. ammon species may have contributed to the genomes of modern domestic sheep.
In many ways, the manuscript is fairly routine, in the sense that the authors describe a shotgun sequencing experiment, subsequent assembly of the data and some comparative analyses of Marco Polo sheep and domesticated ovids. The work appears to have been conducted to a high standard (I lack the expertise to really judge the assembly and annotation methodologies), and it certainly looks to be thorough. It is notable that considerably more sequence data were collected (>1000 Gb) than for the assembly of the domestic sheep genome, where ~220 Gbp and ~155 Gbp of two Texel sheep was generated (Jiang et al. 2014, Science 344:1168). The N50 contig length is a little lower than that of the domestic sheep genome (~30Kb and ~40Kb respectively). Overall, the manuscript contains an impressive amount of data, it is well written, and the resource will be of interest to animal geneticists.
Reply: We are grateful for these positive comments. The N50 contig length of NGS genome assembly could be influenced by many factors, including the strategies of library construction, gap-filling methods and the coverage of Pair-end (PE) libraries (usually with an insert length less than 2kb). There is not much difference between the amount of PE reads used in the assembly and gap-filling of Macro Polo sheep (425.29 Gb clean data) and domestic sheep (374.8 Gb of Illumina sequencing data but with 9 Gb of Roche 454 long sequencing reads data). The N50 contig length of the Marco Polo sheep is slightly shorter than that of published domestic sheep perhaps because the domestic sheep genome project used long reads Roche 454 data, which is now not available in the market. Besides the N50 contig length of 22 NGS mammalian assemblies ranges from 16 to 66 Kb (Table S3 of Wang et al. 2017, GigaScience 6 (4), 1-5), indicating the N50 contig length we obtained here is fairly within the expectation for a mammalian species.
Unsurprisingly, most of the comparisons between Marco Polo sheep and domestic sheep show very high conservation of synteny, GC content, gene number, etc. Given that these analyses are conducted independently of those done in domestic sheep, the similarity can be seen as reassurance that the analyses conducted on Marco Polo sheep genome are robust and reliable. One slight discrepancy is the average intron length (Table S13); in Marco Polo sheep this appears to be ~20% larger than is seen in domestic sheep, goats and cattle. Of course, this could be a real feature of the Marco Polo sheep genome. The authors argue that intron length appears to be conserved

Powered by Editorial Manager® and ProduXion Manager® from Aries Systems Corporation

	across the mammals they study (lines 184-185), but it might be worth trying to establish why it appears to be longer in Marco Polo Sheep.<br>Reply: We thank the reviewer for raising this important issue. We checked the differences of intron length, exon length, etc. between Macro Polo sheep and domestic sheep, goat or human for each orthologous gene. Generally, these parameters were very close within these species (Fig. S7). The increasing of intron length in Macro Polo sheep was not very significant but did existed. More LINE sequences were found in the intron regions of Macro Polo sheep than sheep or goat, suggesting that transposon insertion might have contributed to intron length increase. We have added this discussion on page 10 highlighted with yellow.<br>Table S20 is useful because it presents a list of genes that are putatively under positive selection. It's a bit of a shame that these are not examined or even discussed in the context of adaptation to high altitude, especially as in the Conclusion the authors comment that the genome was partially sequenced because the species is a model for studying this very question. I understand that the primary aim of the paper is to describe the resource, but nonetheless, the results presented in Table S20 warrant some discussion.<br>Reply: Thank you for your suggestion, we have added a detailed paragraph on page 14 to describe the possible biological relevance of positively selected genes in high altitude adaptation, highlighted with yellow.<br>On lines 142-146, the relatively low nucleotide diversity of Marco Polo sheep relative to that seen in domestic sheep is discussed. However, the sampled male was bred and reared in a zoo, and very little is said about whether the diversity is likely to be similar to that seen in wild animals. For example, if the male was inbred, because there were relatively few unrelated captive animals, then the low diversity could be unrepresentative. If the authors know that the animal is not inbred, it could be worth them stating so. Inbreeding would be identifiable through e.g. Runs of Homozygosity.<br>Reply: We fully agree with the referee. The sampled male was originally captured from wild. However, the current available methods, such like Plink, bcftools, are unworkable for only one individual. Thus, we made a rough inference of RoH based on the distribution of heterozygosity along the genome with Hidden Markov model. We found the RoH regions (heterozygosity ratio < 0.003%, 1/46 of average) were made up 14 % of the total 49,317 non-overlapping 50k window. It should be noted that this result should be interpreted with caution because this is only from one individual. We have described more about this issue in the revised manuscript highlighted with yellow.<br>Minor typos:<br>1) Line 405 - 'Veen' diagram should be 'Venn'.<br>Reply: Sorry for the typo. Corrected as suggested.
**Additional Information:**	
**Question**	**Response**
Are you submitting this manuscript to a special series or article collection?	No
**Experimental design and statistics**<br><br>Full details of the experimental design and statistical methods used should be given in the Methods section, as detailed in our Minimum Standards Reporting Checklist. Information essential to interpreting the data presented should be made available in the figure legends.<br><br>Have you included all the information requested in your manuscript?	Yes
**Resources**	Yes

A description of all resources used, including antibodies, cell lines, animals and software tools, with enough information to allow them to be uniquely identified, should be included in the Methods section. Authors are strongly encouraged to cite Research Resource Identifiers (RRIDs) for antibodies, model organisms and tools, where possible.<br><br>Have you included the information requested as detailed in our Minimum Standards Reporting Checklist?	
**Availability of data and materials**<br><br>All datasets and code on which the conclusions of the paper rely must be either included in your submission or deposited in publicly available repositories (where available and ethically appropriate), referencing such data using a unique identifier in the references and in the "Availability of Data and Materials" section of your manuscript.<br><br>Have you have met the above requirement as detailed in our Minimum Standards Reporting Checklist?	Yes

Powered by Editorial Manager® and ProduXion Manager® from Aries Systems Corporation

Manuscript

Click here to download Manuscript Marco polo sheep-MS-revised.docx

Draft genome of the Marco Polo Sheep (*Ovis ammon polii*)

Yongzhi Yang[1,†], Yutao Wang[2,3,†], Yue Zhao[4†], Xiuying Zhang[2,3], Ran Li[4], Lei Chen[1], Guojie Zhang[5], Yu Jiang[4], Qiang Qiu[1], Wen Wang[1*], Hongjiang Wei[6*], Kun Wang[1,*]

[1] Center for Ecological and Environmental Sciences, Northwestern Polytechnical University, Xi'an 710072, China

[2] College of Life and Geographic Sciences, Kashgar University, Kashgar 844000, China

[3] The Key Laboratory of Ecology and Biological Resources in Yarkand Oasis at Colleges & Universities under the Department of Education of Xinjiang Uygur Autonomous Region, Kashgar University, Kashgar 844000, China

[4] College of Animal Science and Technology, Northwest A&F University, Yangling 712100, China

[5] Centre for Social Evolution, Department of Biology, Universitetsparken 15, University of Copenhagen, Copenhagen 2100, Denmark

[6] Key Laboratory of Banna Miniature Inbred Pig of Yunnan Province, College of Animal Science and Technology, Yunnan Agricultural University, Kunming 650225, China

[*] Correspondence: wk8910@gmail.com (KW), hongjiangwei@126.com (HW), wwang@wangweb-lab.org or wwang@mail.kiz.ac.cn (WW)

[†]These authors contributed equally to this work.

Abstract

Background: The Marco Polo Sheep (*Ovis ammon polii*), a subspecies of argali (*Ovis ammon*) which is distributed mainly in the Pamir Mountains, provides a mammalian model in which to study high-altitude adaptation mechanisms. Due to over-hunting and subsistence poaching, as well as competition with livestock and habitat loss, *O. ammon* has been categorized as an endangered species on several lists. It can have fertile offspring with sheep. Hence a high quality reference genome of the Marco Polo Sheep will be very helpful in conservation genetics and even in exploiting useful genes in sheep breeding.

Findings: A total of 1,022.43 Gb of raw reads resulting from whole-genome sequencing of a Marco Polo Sheep were generated using an Illumina HiSeq2000 platform. The final genome assembly (2.71 Gb), which has an N50 contig size of 30.7 Kb and a scaffold N50 of 5.49 Mb. The repeat sequences identified account for 46.72% of the genome and 20,336 protein-coding genes were predicted from the masked genome. Phylogenetic analysis indicated a close relationship between Marco Polo Sheep and the domesticated sheep, and the time of their divergence was approximately 2.36 million years ago (Mya). We identified 271 expanded gene families and 168 putative positively selected genes in the Marco Polo Sheep lineage.

Conclusions: We provide the first genome sequence and gene annotation for the Marco Polo Sheep. The availability of these resources will be of value in the future conservation of this endangered large mammal, for research into high-altitude adaptation mechanisms, for reconstructing the evolutionary history of the *Caprinae*

and for the future conservation of the Marco Polo Sheep.

Keywords: Marco Polo Sheep, genome assembly, annotation, evolution.

Data description

Introduction to *O. ammon polii*

The Marco Polo Sheep (*Ovis ammon polii*) is a subspecies of argali (*Ovis ammon*), named after the explorer Marco Polo and was first described scientifically in 1841 by Edward Blyth [1]. This subspecies is distributed mainly in the Pamir Mountains, which consist of rugged ranges at elevations of 3,500-5,200 m [2]. The habitat of the subspecies includes the Tajikistan Pamir Mountains [3], as well as in limited regions in China, Afghanistan, Pakistan, and Kyrgyzstan [4]. The Marco Polo Sheep species represents a new model in which to study high-altitude adaptation mechanisms adopted by mammals. Due to the sheep's impressively long horns, foreign hunters have for many years been willing to pay large amounts of money to take part in a hunt [5] and this is still the case today [2]. Recent studies on the status of the argali population have shown a decline in numbers, caused mainly by over-hunting and subsistence poaching, as well as by competition with livestock and habitat loss [6-9]. *O. ammon* has been categorized in several protection lists, such as Appendix II of CITES (Convention on International Trade in Endangered Species of Wild Fauna and Flora) and the IUCN (International Union for Conservation of Nature and Natural Resources) Red List, as a vulnerable or near threatened species. Conservation and restoration measures are therefore needed in order to safeguard the species, and information about its genome will be a key element in formulating an appropriate conservation strategy.

Sequencing

High molecular weight genomic DNA was extracted from fibroblast cells cultured from the ear skin biopsy sample of a male *O. ammon polii* using a Qiagen DNA purification kit. The sheep was originally captured from the Pamir Plateau of China and reared in the KaShi Zoo, Kashgar Prefecture, Xinjiang Province, China. A whole-genome shotgun sequencing strategy was applied, and a series of libraries with insert seizes ranging from 400 base pairs (bp) to 15 kilobase pairs (kb) were constructed using the standard protocol provided by Illumina (San Diego, CA, USA). To construct small-insert libraries (400, 500, 600, 700 and 800 bp), DNA was sheared to the target size range using a Covaris S2 sonicator (Covaris, Woburn, MA, USA) and ligated to adaptors. For long-insert libraries (4, 8, 10, 12 and 15 kb), DNA was fragmented using a Hydroshear system (Digilab, Marlborough, MA, USA). Sheared fragments were end-labelled with biotin and fragments of the desired size were gel purified. A second round of fragmentation was then conducted before adapter ligation. All libraries were sequenced on an Illumina HiSeq 2000 platform (**Table S1**). A total of 1,022.43 Gb of raw data was generated, and 624.74 Gb of clean data was retrieved after removal of duplicates, contaminated reads (reads with adaptor sequence) and low quality reads using the sickle software tool (https://github.com/najoshi/sickle) with a quality threshold of 10 and a length threshold of 50. We further corrected the short-insert library reads using SOAPec [10], a k-mer-based error correction package.

Evaluation of genome size

Approximately 65 Gb clean reads were randomly selected from all short libraries to estimate the genome size using the k-mer-based method and the formula: G = k-mer_number/k-mer_depth. In this study, a total of 52,413,427,492 k-mers were generated and the peak k-mer depth was 17. The genome size was estimated to be approximately 3 Gb (**Table S2** and **Fig. S1**) and all the clean data correspond to a coverage of ~ 208-fold.

De novo genome assembly

The assembly was performed using Platanus v1.2.4 [11], which is well suited to high-throughput short reads and heterozygous diploid genomes. Briefly, error-corrected paired-end reads (insert size < 2 kb) were input for contig assembly with the default parameters. Next, all cleaned paired-end (insert size < 2 kb) reads and mate-paired (insert size > 2 kb) reads were mapped onto the contigs for scaffold building, using default parameters except that the minimum number of links (-l) was set to 10 in order to minimize the number of scaffolding errors. After gap filling by Platanus, the gaps that still remained in the resulting scaffolds were closed using GapCloser [10]. The final *de novo* assembly for the Marco Polo Sheep has a total length of 2.71 Gb, including 116.91 Mb (4.3 %) unknown bases. The assembly is slightly larger than that of the domestic sheep (*Ovis aries*, **Oar_v3.1**, 2.61 Gb) [12] and smaller than that of the domestic goat (*Capra hircus*, **ARS1**, 2.92 Gb) [13]. The N50s for contigs and scaffolds of the Marco Polo Sheep genome are, respectively, 30.8 kb and 5.5 Mb (**Table S3**). The assembled scaffolds represented ~ 88% of the

estimated genome size, and the GC content was 41.9%, similar to those of sheep (41.9%) and goat (41.5%) (**Fig. S2**).

We assessed the quality of the genome assembly with respect to base-level accuracy, integrity, and continuity. More than 99.65% of the short insert paired-end reads could be mapped to the assembly and more than 98.35% of the sequence have a coverage depth greater than 20-fold (**Table S4**), thus the assembly is of high level of single-base accuracy. A core eukaryotic genes (CEG) mapping approach (CEGMA, v2.5 [14]) dataset comprising 248 CEGs was used to evaluate the completeness of the draft: 93.55% (232/248) of genes were completely or partially covered in the assembled genome (**Table S5**). Alongside this, we also used the BUSCO v2.0.1 [15] (the representative mammal gene set mammalia_odb9, which contains 4,104 single-copy genes that are highly conserved in mammals) software package to assess the quality of the genome assembly generated. The resulting BUSCO value was 95.9%, containing C: 92.5% [S: 91.3%, D: 1.2%], F: 3.4%, M: 4.1%, n: 4104 (C: complete [D: duplicated], F: fragmented, M: missed, n: genes) (**Table S6**). Both the CEGMA and the BUSCO scores are comparable to those for sheep (Oar_v3.1) and domestic goat (ARS1 and CHIR_1.0), which are known for their high quality as the references genomes of two important livestock animals, suggesting our Marco Polo Sheep assembly is of high quality and quite complete. Finally, to evaluate the trade-off between the contiguity and correctness of our assembly, we applied the feature-response curve (FRC) method [16], which predicts the correctness of an assembly by identifying ‘features’ representing potential errors or complications on

each *de novo* assembled scaffold during the assembly process. The FRC curve was calculated for the Marco Polo Sheep, sheep, taurine cattle and two versions of goat assemblies (**Fig. S3**). We found that the curve for our assembly was similar to that for the sheep and the two goat assemblies, with taurine cattle slightly different from the others, indicating the level of contiguity and correctness of the Marco Polo Sheep genome assembly is comparable to those of sheep and goat.

We mapped the reads from short-insert length libraries to the Marco Polo Sheep reference genome with BWA [17] and performed variant calling with SAMtools v0.1.19 [18]. Applying strict quality control and filtering, we obtained a total of 3.5 million SNVs (**Table S7**) and noted that the heterozygosity rate (0.14 %) was lower than that estimated for sheep (**Oar_v3.1**, 0.2 %) and similar with that of goat (**CHIR_1.0**, 0.13 %) [12]. We further assessed the distribution of heterozygosity ratio of non-overlapping 50K windows (Fig. S4). We assume that the heterozygosity on the genome can be divided into three states (low/normal/high) and applied Hidden Markov model with depmixS4 package [19] in R to infer the state of each window. The windows with “low heterozygosity” state (0.003%), made up 14% of the genome, were almost completely homogeneous, which is obvious feature of endangered animals [20]. It should be noted that this result should be interpreted with caution because this is only from one individual. 156 genes were overlap of more than half length with the low heterozygosity regions and the GO enrichment analysis shows that no GO category was significant enriched (Table S8). A total of 384,018 insertions and deletions (InDels) (**Table S9**) were obtained. Similar to the findings of previous

studies on yak [21] and wisent [22], the InDels in the coding regions were enriched for sizes that are multiples of three bases (**Fig. S5**).

Annotation

The transposable elements present in Marco Polo sheep sequences were identified using a combination of *de novo* and homology-based approaches. Transposable elements were identified at both the DNA and the protein levels, based on known sequences contained within the DNA repeat database (RepBase v21.01) [23], using RepeatMasker (v4.0.5) [24] and RepeatProteinMask (v4.0.5, a package within RepeatMasker). For the *de novo* prediction, firstly RepeatModeler (V1.0.8, http://www.repeatmasker.org/RepeatModeler) was employed to construct a *de novo* repeat library, then RepeatMasker was used to identify repeats using both the *de novo* repeat database and RepBase. We then combined the *de novo* prediction and the homolog prediction of transposable elements according to the coordination in the genome. Tandem repeats were annotated with RepeatMasker and Tandem Repeats Finder (TRF, V4.07) [25]. In summary, a total of 0.87% tandem repeats and 46.60% transposable elements were identified in the Marco Polo sheep assembly, with LINEs constituting the greatest proportion, 72.48% of all repeats, and SINEs making up 24.09% of all repeats (**Table S10** and **Table S11**).

We used homology-based and *de novo* prediction to annotate protein coding genes. For homology-based prediction, protein sequences from 5 different species (*Bos taurus*, *Equus caballus*, *Homo sapiens*, *Ovis aries*, *Sus scrofa*) (**Table S12**) were

mapped onto the repeat-masked Marco Polo sheep genome using TblastN with an E-value cutoff of 1e-5; the aligned sequences as well as the corresponding query proteins were then filtered and passed to GeneWise [26] to search for accurately spliced alignments. For *de novo* prediction, we first randomly selected 1500 full-length genes from the results of homology-based prediction to train the model parameters for Augustus v3.2.1 [27] and geneid v1.4.4 [28]. GenScan [29], Augustus v3.2.1 [27] and geneid v1.4.4 [28] were then used to predict genes based on the training set of human and Marco Polo Sheep genes. We used EVidenceModeler software (EVM, version 1.1.1) to integrate the genes predicted by the homology and *de novo* approaches and generated a consensus gene set (Table S13). The final gene set was produced by removing low-quality genes of short length (proteins with fewer than 50 amino acids) and/or exhibiting premature termination. The final total gene set consisted of 20,336 genes, and the number of genes, gene length distribution and exon number per gene were similar to those of other mammals, while the intron length was slightly larger than goat (CHIR_1.0), sheep (Oar_v3.1) and taurine cattle (UMD3.1) (Table S14 and Fig. S6, S7). The repeat content was annotated by RepeatMasker (v4.0.5) [24] with unified parameters for Macro Polo sheep, domestic sheep and goat. We found that there were more LINE sequences in the intron regions of Marco Polo sheep than the other species, suggesting that transposon insertions might have contributed to intron length increasing (Fig. S8). 92.55% of all the predicted genes could be annotated using five protein databases: InterPro (87.17%), GO (Gene ontology, 70.99%), Swiss-Prot (91.67%), TrEMBL (92.33%) and KEGG (Kyoto

Encyclopedia of Genes and Genomes, 57.25%) (**Table S15**). In addition, we identified 2,978 noncoding RNAs in the Marco Polo Sheep genome (**Table S16**).

Genome evolution

Firstly, large-scale variations among Marco Polo Sheep, sheep and goat were identified by the synteny analysis using the program LAST [30]. A total of 2.29/2.30/2.40 Gb 1:1 alignment sequences were generated for, respectively Marco Polo Sheep vs sheep (Oar_v3.1), Marco Polo Sheep vs goat (ASR1), sheep vs goat, covering more than 88.55% of each genome (**Table S17** and **Fig. S9**). The sequences present on sheep/goat autosomes were well covered (average values: 89.65%/89.88%) by the synteny alignment, whereas only 66.09%/63.03% were covered in the case of chromosome X. The scaffolds of the Marco Polo Sheep genome that aligned to the sex chromosomes were also more fragmented. The divergence between Marco Polo Sheep vs sheep (Oar_v3.1), Marco Polo Sheep vs goat (ASR1), sheep vs goat was 0.7%, 2.2%, 2.3%, respectively, corresponding to their relatedness (**Table S17** and **Fig. S10**). Although Marco Polo Sheep, sheep and goat showed good synteny alignments, there are large numbers of inter-chromosomal rearrangements between pairs of them (**Fig. S11** and **S12**). By comparing Marco Polo Sheep and sheep/goat genomes we identified 11,756/6,026 inter-chromosomal, intra-chromosomal, or inversion breakpoints (edges of transposition events) (**Table S18**), which may have been caused by the real translocations events between them as they have a different karyotype, errors in the assembly of the genomes or erroneous synteny alignments

(false positives and false negatives). However, at this stage it is difficult to distinguish between possible artifactual and real effects. The breakpoint distributions were significantly enriched in repeat regions (**Fig S13a**), which are susceptible to rearrangements but also to assembly or alignment errors. Longer scaffolds were found to harbor fewer breakpoints (**Fig. S13b)**. Single molecule sequencing with unbiased long reads will be the best way of identifying large-scale variation.

To analyze gene families, we downloaded the protein sequences of eight additional species (Opossum, human, dog, horse, pig, taurine cattle, goat and sheep) from Ensembl [31] and GigaDB [32] (**Table S12**). The consensus gene set for the above eight species and Marco Polo Sheep were filtered to retain the longest CDS (coding sequence) for each gene, removing CDS with premature stop codons and those protein sequences < 50 amino acids in length, resulting in a dataset of 188,359 protein sequences, which was used as the input file for OrthoMCL [33]. A total of 17,578 OrthoMCL families were built utilizing an effective database size of all-to-all BLASTP strategy with an E-value of 1e-5 and a Markov Chain Clustering default inflation parameter (**Table S19** and **Fig. 1a**). We identified 155 gene families that were specific to the Marco Polo Sheep when comparing with taurine cattle, sheep, goat and horse (**Fig. 1b**), and detected 271 gene families that have expanded in the Marco Polo Sheep lineage using CAFÉ (Computational Analysis of gene Family Evolution, v4.0.1) [34] **(Fig. 1a**). The expanded gene families were enriched in 38 GO categories and their functions were mainly associated with response to stimulus, cell adhesion, G-protein coupled receptor and enzyme activity (**Table S20**).

Next, we selected 5,788 single-copy gene families from the above-mentioned 9 mammalian species and used PRANK v3.8.31 [35] with the codon option to align the CDS from each single-copy gene family. 4D-sites (fourfold degenerate sites) were extracted from all the single-copy genes and used to construct a phylogenetic tree with the GTR+G+I model in RAxML v7.2.8 [36] (**Fig. S14**). The divergence time of each node was estimated by the PAML MCMCtree program v4.5 [37] and calibrated against the timing of the divergence of the opossum and human (124.6-134.8 Mya), human and taurine cattle (95.3-113 Mya), taurine cattle and pig (48.3-53.5 Mya), and taurine cattle and goat (18.3-28.5 Mya) [38]. The convergence was checked by Tracer v1.5 [39] and confirmed by two independent runs. The phylogenetic analysis showed that the Marco Polo Sheep has a closer relationship with sheep than with other mammals and that the divergence time between them is about 2.36 (1.94-2.61) Mya (**Fig. 1a**).

We further used the free ratio model to calculate the average Ka/Ks values and the branch-site likelihood ratio test to identify positively selected genes in the Marco Polo Sheep lineage. A total of 10,353 high confidence single-copy genes were identified by InParanoid and MultiParanoid within the human, dog, taurine cattle, goat, sheep and Marco Polo Sheep. We found that the Marco Polo Sheep has a regular level of the average Ka/Ks values, but containing more outliers (**Fig. 1c**). A total of 168 positively selected genes were identified in the Marco Polo Sheep lineage (Table S21), and six of them were orthologous with high altitude adaptation related genes (IDE, IGF1, P2RX3, PHF6, PROX1 and RYR1) identified in Tibet wild boar [40]. Two

genes were associated with hypoxia response: the ryanodine receptor protein encoded by *RYR1* (Ryanodine Receptor 1) was located in the pulmonary artery smooth muscle cells, which could subserve coupled O_2 sensor and NO regulatory functions to response to the tissue hypoxic decrease [41]; *P2RX3* (Purinergic Receptor P2X, Ligand-Gated Ion Channel, 3), is reported as a potential new target for the control of human hypertension, which could reduce the arterial pressure and basal sympathetic activity and normalize carotid body hyperreflexia in conscious rats with hypertension during P2RX3 antagonism [42]. Four genes were related with energetic metabolism: *IGF1* (Insulin-like Growth Factor 1) encodes the growth-promoting polypeptide mainly involved in the body growth and differentiation and as well as the glucose, lipid and protein metabolism [43]; *IDE* (Insulin Degrading Enzyme) encodes a zinc metallopeptidase that degrades intracellular insulin, which could accelerates glycolysis, pentose phosphate cycle, and glycogen synthesis in liver [44]; *PHF6* (PHD Finger Protein 6) encodes a protein with two PHD-type zinc finger domains and its function was associated with Börjeson-Forssman-Lehmann syndrome, which is one of the syndromic obesities in humans [45]; the protein encoded by *PROX1* (Prospero Homeobox 1) could occupy promoters of metabolic genes on a genome-wide scale to control of energy homeostasis [46]. In addition, the other identify PSGs may also be associated to high altitude adaptation, while there are rare literature data on the function of them. Further studies will be required to clarify the roles of these genes in high altitude tolerance.

Finally, we inferred the demographic history of the Marco Polo Sheep using the

Pairwise Sequentially Markovian Coalescent (PSMC) model [47]. Consensus sequences were obtained using SAMtools v0.1.19 [18] and divided into non-overlapping 100 bp bins. The analysis was performed with the following parameters: -N25 -t15 -r5 -p '4+25×2+4+6'. PSMC modeling was done using a bootstrapping approach, with sampling performed 100 times to estimate the variance of the simulated results. The effective population size (N_e) of Marco Polo Sheep shows a peak at ~1 Mya followed by two distinct declines. The most recent decline involved at least a sevenfold decrease in N_e, and occurred ~ 60,000 years ago (Fig. S15).

Conclusion

In summary, the novel genome data generated in this work will provide a valuable resource for studying high-altitude adaptation mechanisms within mammals and for investigating the evolutionary histories of the *Caprinae*, and it will have relevance for the future conservation of the Marco Polo Sheep.

Availability of supporting data

The sequencing reads of each sequencing library have been deposited at NCBI with the Project ID: PRJNA391748, Sample ID: SAMN07274464, and the Genome Sequence Archive [48] in BIG Data Center [49], Beijing Institute Genomics (BIG), Chinese Academy of Science, under accession number PRJCA000449 that are publicly accessible at http://bigd.big.ac.cn/gsa. The assembly and annotation of the

Marco Polo Sheep genome are available in the *GigaScience* GigaDB database. Supplementary figures and tables are provided in Additional file 1.

Competing interests

The authors declare that they have no competing interests.

Authors' contributions

KW and WW conceptualized the research project. KW, WW and HW designed analytic strategy and coordinated the project. YW, HW and WW collected the samples and led the genome sequencing. YY and KW led the bioinformatics analysis. YY, YW and YZ generated the genome assembly and the genome annotation. YY, RL and LC finished the synteny analysis. YW, YZ and GZ performed the gene family construction and the phylogeny analysis. YY and QQ detected the PSGs and carried out data submission. YY, WW and KW wrote the paper. All authors read and approved the final manuscript.

Acknowledgements

This study was supported by research grants from the National Natural Science Foundation of China (No. 31072019 and No. 31572381), and Talents Team Construction Fund of Northwestern Polytechnical University (NWPU) to QQ and WW. We thank Nowbio Biotech Inc., Kunming, China for the remarkable work on DNA libraries constructions and the assistance during the genome sequencing.

References

1. Dohner JV. The encyclopedia of historic and endangered livestock and poultry breeds. Yale University Press. 2001:p. 514.
2. Schaller GB and Kang A. Status of Marco Polo sheep *Ovis ammon polii* in China and adjacent countries: conservation of a Vulnerable subspecies. Oryx. 2008;42 1:100-6. doi:10.1017/S0030605308000811.
3. Breu TMH, Hans The Tajik Pamirs: Challenges of sustainable development in an isolated mountain region. Centre for Development and Environment (CDE), University of Berne: Berne, Switzerland. 2003:p. 80.
4. Valdez R, Michel S, Subbotin A and Klich D. Status and population structure of a hunted population of Marco Polo Argali *Ovis ammon polii* (Cetartiodactyla, Bovidae) in Southeastern Tajikistan. Mammalia. 2016;80 1:49-57. doi:10.1515/mammalia-2014-0116.
5. Harris RB. Ecotourism versus trophy hunting: incentives toward conservation in Yeniugou, Tibetan Plateau, China. Integrating People and Wildlife for a Sustainable Future (eds JA Bissonette & PR Krausman). 1995:228-34.
6. Harris RB and Reading R. *Ovis ammon.* The IUCN Red List of Threatened Species 2008: e.T15733A5074694. http://dx.doi.org/10.2305/IUCN.UK.2008.RLTS.T15733A5074694.en. Downloaded on 02 May 2017. 2008.
7. Shackleton DM. Wild sheep and goats and their relatives. 1997.
8. Nowak R. Court upholds controls on imports of argali trophies. Endangered Species Technical Bulletin. 1993;18 4:11-2.
9. Shrestha R and Wegge P. Wild sheep and livestock in Nepal Trans-Himalaya: coexistence or competition? Environmental Conservation. 2008;35 02:125-36.
10. Luo R, Liu B, Xie Y, Li Z, Huang W, Yuan J, et al. SOAPdenovo2: an empirically improved memory-efficient short-read de novo assembler. Gigascience. 2012;1 1:18. doi:10.1186/2047-217X-1-18.
11. Kajitani R, Toshimoto K, Noguchi H, Toyoda A, Ogura Y, Okuno M, et al. Efficient de novo assembly of highly heterozygous genomes from whole-genome shotgun short reads. Genome Res. 2014;24 8:1384-95. doi:10.1101/gr.170720.113.
12. Jiang Y, Xie M, Chen WB, Talbot R, Maddox JF, Faraut T, et al. The sheep genome illuminates biology of the rumen and lipid metabolism. Science. 2014;344 6188:1168-73. doi:10.1126/science.1252806.
13. Bickhart DM, Rosen BD, Koren S, Sayre BL, Hastie AR, Chan S, et al. Single-molecule sequencing and chromatin conformation capture enable de novo reference assembly of the domestic goat genome. Nat Genet. 2017;49 4:643-50. doi:10.1038/ng.3802.
14. Parra G, Bradnam K, Ning Z, Keane T and Korf I. Assessing the gene space in draft genomes. Nucleic Acids Res. 2009;37 1:289-97. doi:10.1093/nar/gkn916.
15. Simao FA, Waterhouse RM, Ioannidis P, Kriventseva EV and Zdobnov EM. BUSCO: assessing genome assembly and annotation completeness with

single-copy orthologs. Bioinformatics. 2015;31 19:3210-2. doi:10.1093/bioinformatics/btv351.

16. Vezzi F, Narzisi G and Mishra B. Reevaluating assembly evaluations with feature response curves: GAGE and assemblathons. PLoS One. 2012;7 12:e52210. doi:10.1371/journal.pone.0052210.

17. Li H. Aligning sequence reads, clone sequences and assembly contigs with BWA-MEM. arXiv preprint arXiv:13033997. 2013.

18. Li H, Handsaker B, Wysoker A, Fennell T, Ruan J, Homer N, et al. The Sequence Alignment/Map format and SAMtools. Bioinformatics. 2009;25 16:2078-9. doi:10.1093/bioinformatics/btp352.

19. Visser I, Speekenbrink M: depmixS4: An R-package for hidden Markov models. *Journal of Statistical Software* 2010, 36(7):1-21.

20. Dobrynin P, Liu S, Tamazian G, Xiong Z, Yurchenko AA, Krasheninnikova K, Kliver S, Schmidt-Kuntzel A, Koepfli KP, Johnson W *et al*: Genomic legacy of the African cheetah, Acinonyx jubatus. *Genome Biol* 2015, 16:277.

21. Qiu Q, Zhang G, Ma T, Qian W, Wang J, Ye Z, et al. The yak genome and adaptation to life at high altitude. Nat Genet. 2012;44 8:946-9. doi:10.1038/ng.2343.

22. Wang K, Wang L, Lenstra JA, Jian J, Yang Y, Hu Q, et al. The genome sequence of the wisent (*Bison bonasus*). Gigascience. 2017; doi:10.1093/gigascience/gix016.

23. Bao W, Kojima KK and Kohany O. Repbase Update, a database of repetitive elements in eukaryotic genomes. Mob DNA. 2015;6:11. doi:10.1186/s13100-015-0041-9.

24. Tarailo-Graovac M and Chen N. Using RepeatMasker to identify repetitive elements in genomic sequences. Curr Protoc Bioinformatics. 2009;Chapter 4:Unit 4 10. doi:10.1002/0471250953.bi0410s25.

25. Benson G. Tandem repeats finder: a program to analyze DNA sequences. Nucleic Acids Res. 1999;27 2:573-80.

26. Birney E, Clamp M and Durbin R. GeneWise and Genomewise. Genome Res. 2004;14 5:988-95. doi:10.1101/gr.1865504.

27. Stanke M, Diekhans M, Baertsch R and Haussler D. Using native and syntenically mapped cDNA alignments to improve de novo gene finding. Bioinformatics. 2008;24 5:637-44. doi:10.1093/bioinformatics/btn013.

28. Blanco E, Parra G and Guigo R. Using geneid to identify genes. Current protocols in bioinformatics. 2007;Chapter 4:Unit 4.3. doi:10.1002/0471250953.bi0403s18.

29. Burge CB and Karlin S. Finding the genes in genomic DNA. Curr Opin Struct Biol. 1998;8 3:346-54.

30. Kielbasa SM, Wan R, Sato K, Horton P and Frith MC. Adaptive seeds tame genomic sequence comparison. Genome Res. 2011;21 3:487-93. doi:10.1101/gr.113985.110.

31. Yates A, Akanni W, Amode MR, Barrell D, Billis K, Carvalho-Silva D, et al. Ensembl 2016. Nucleic Acids Research. 2016;44 D1:D710-D6.

doi:10.1093/nar/gkv1157.
32. Dong Y, Xie M, Jiang Y, Xiao NQ, Du XY, Zhang WG, et al. Genomic data of the domestic goat (*Capra hircus*). GigaScience Database http://dxdoiorg/105524/100082. 2013.
33. Li L, Stoeckert CJ, Jr. and Roos DS. OrthoMCL: identification of ortholog groups for eukaryotic genomes. Genome Res. 2003;13 9:2178-89. doi:10.1101/gr.1224503.
34. De Bie T, Cristianini N, Demuth JP and Hahn MW. CAFE: a computational tool for the study of gene family evolution. Bioinformatics. 2006;22 10:1269-71. doi:10.1093/bioinformatics/btl097.
35. Loytynoja A and Goldman N. An algorithm for progressive multiple alignment of sequences with insertions. Proc Natl Acad Sci U S A. 2005;102 30:10557-62. doi:10.1073/pnas.0409137102.
36. Stamatakis A. RAxML version 8: a tool for phylogenetic analysis and post-analysis of large phylogenies. Bioinformatics. 2014;30 9:1312-3. doi:10.1093/bioinformatics/btu033.
37. Yang Z. PAML 4: phylogenetic analysis by maximum likelihood. Mol Biol Evol. 2007;24 8:1586-91. doi:10.1093/molbev/msm088.
38. Benton MJ and Donoghue PC. Paleontological evidence to date the tree of life. Mol Biol Evol. 2007;24 1:26-53. doi:10.1093/molbev/msl150.
39. Rambaut A and Drummond A. Tracer v1. 5 Available from http://beast. bio. ed. ac. uk/Tracer. Accessed, 2013.
40. Li M, Tian S, Jin L, Zhou G, Li Y, Zhang Y, Wang T, Yeung CK, Chen L, Ma J *et al*: Genomic analyses identify distinct patterns of selection in domesticated pigs and Tibetan wild boars. *Nat Genet* 2013, 45(12):1431-1438.
41. Wang YX, Zheng YM: ROS-dependent signaling mechanisms for hypoxic Ca(2+) responses in pulmonary artery myocytes. *Antioxid Redox Signal* 2010, 12(5):611-623.
42. Pijacka W, Moraes DJ, Ratcliffe LE, Nightingale AK, Hart EC, da Silva MP, Machado BH, McBryde FD, Abdala AP, Ford AP: Purinergic receptors in the carotid body as a new drug target for controlling hypertension. *Nature* 2016, 201:6.
43. Cai WK, Sakaguchi M, Kleinridders A, Gonzalez-Del Pino G, Dreyfuss JM, O'Neill BT, Ramirez AK, Pan H, Winnay JN, Boucher J *et al*: Domain-dependent effects of insulin and IGF-1 receptors on signalling and gene expression. *Nat Commun* 2017, 8.
44. Rudovich N, Pivovarova O, Fisher E, Fischer-Rosinsky A, Spranger J, Mohlig M, Schulze MB, Boeing H, Pfeiffer AF: Polymorphisms within insulin-degrading enzyme (IDE) gene determine insulin metabolism and risk of type 2 diabetes. *J Mol Med (Berl)* 2009, 87(11):1145-1151.
45. Chung WK, Leibel RL: Molecular physiology of syndromic obesities in humans. *Trends Endocrinol Metab* 2005, 16(6):267-272.
46. Charest-Marcotte A, Dufour CR, Wilson BJ, Tremblay AM, Eichner LJ, Arlow DH, Mootha VK, Giguere V: The homeobox protein Prox1 is a negative

modulator of ERRα/PGC-$_{1\alpha}$ bioenergetic functions. *Genes Dev* 2010, 24(6):537-542.

47. Li H, Durbin R: Inference of human population history from individual whole-genome sequences. *Nature* 2011, 475(7357):493-496.

48. Wang Y, Song F, Zhu J, Zhang S, Yang Y, Chen T, et al. GSA: Genome Sequence Archive. Genom. Proteom. Bioinform. 2017;15 1:14-8. doi:10.1016/j.gpb.2017.01.001.

49. Members BIGDC. The BIG Data Center: from deposition to integration to translation. Nucleic Acids Res. 2017;45 D1:D18-D24. doi:10.1093/nar/gkw1060.

Figure 1. Phylogenetic relationships and genomic comparisons between Marco Polo Sheep and other mammals. (a) Divergence time estimates for the nine mammals generated using MCMCtree and the 4-fold degenerate sites. The red dots correspond to calibration points and the divergence times. Divergence time estimates (Mya) are indicated above the appropriate nodes; blue nodal bars indicate 95 % confidence intervals. Gene orthology was determined by comparing the genomes with the OrthoMCL software. (b) A Venn diagram of the shared orthologues among Marco Polo Sheep, sheep, goat, taurine cattle and horse. Each number represents a gene family number. (c) The box plot shows the ratio of non-synonymous to synonymous mutations (Ka/Ks) for Marco Polo Sheep, sheep, goat, taurine cattle, horse and human.

Additional files

Figure S1. 21-mer-based analysis carried out to estimate the size of the Marco Polo Sheep genome.

Figure S2. GC content distribution for the genomes of Marco Polo Sheep, goat and sheep.

Figure S3. FRCurve of five genome assemblies.

Figure S4. The distribution of observed heterozgosity stats within Marco Polo Sheep genome.

Figure S5. Counts of InDels in coding regions, showing an enrichment of multiples of three bases.

Figure S6. Comparison of gene structure characteristics with those of other mammals.

Figure S7. Comparison of gene structure characteristics of the 1:1 orthologs in the five mammals.

Figure S8. Comparison of the repeat content in the intron regions among Marco Polo Sheep, Sheep (Oar_v3.1) and Goat (CHIR_1.0).

Figure S9. Summary of the number of chromosomes to which a given scaffold of the Marco Polo Sheep genome could be aligned.

Figure S10. Divergence between Marco Polo Sheep, sheep and goat.

Figure S11. Synteny relationship between Marco Polo Sheep and sheep.

Figure S12. Synteny relationship between Marco Polo Sheep and goat.

Figure S13. Density of breakpoints (number per million bases) in different regions of the genome.

Figure S14. Phylogeny relationships between Marco Polo Sheep and other mammals.

Figure S15. Demographic history of Marco Polo Sheep.

Table S1. Summary of sequenced reads.

Table S2. Estimation of genome size based on 21-mer statistics.

Table S3. Statistics for the final assemblies of the Marco Polo Sheep genome.

Table S4. Numbers of reads mapped to the assembled Marco Polo Sheep genome.

Table S5. Summary of CEGMA analysis results.

Table S6. Summary of BUSCO analysis results obtained by counting matches to 4104 single-copy orthologs (mammalia_odb9).

Table S7. The distribution of SNVs in the Marco Polo Sheep genome.

Table S8. Genes located in the low heterozygosity regions.

Table S9. The distribution of InDels in the wisent genome.

Table S10. Prediction of repetitive elements in the assembled Marco Polo Sheep genome.

Table S11. Classification of interspersed repeats in the assembled Marco Polo Sheep genome.

Table S12. Data on all species used during the genome analysis.

Table S13. Prediction of protein-coding genes in the Marco Polo Sheep.

Table S14. Comparative gene statistics.

Table S15. Functional annotation of predicted genes in the Marco Polo Sheep.

Table S16. Summary statistics of non-coding RNAs in the Marco Polo Sheep.

Table S17. Summary of synteny alignments.

Table S18. Summary of breakpoints between Marco Polo Sheep, sheep and goat.

Table S19. Summary statistics of gene families in 9 species.

Table S20. GO enrichment analysis of the expanded gene families in the Marco Polo Sheep lineage.

Table S21. Candidate positively selected genes (PSGs) in the Marco Polo Sheep lineage.

Figure1
Click here to download Figure Figure 1.pdf

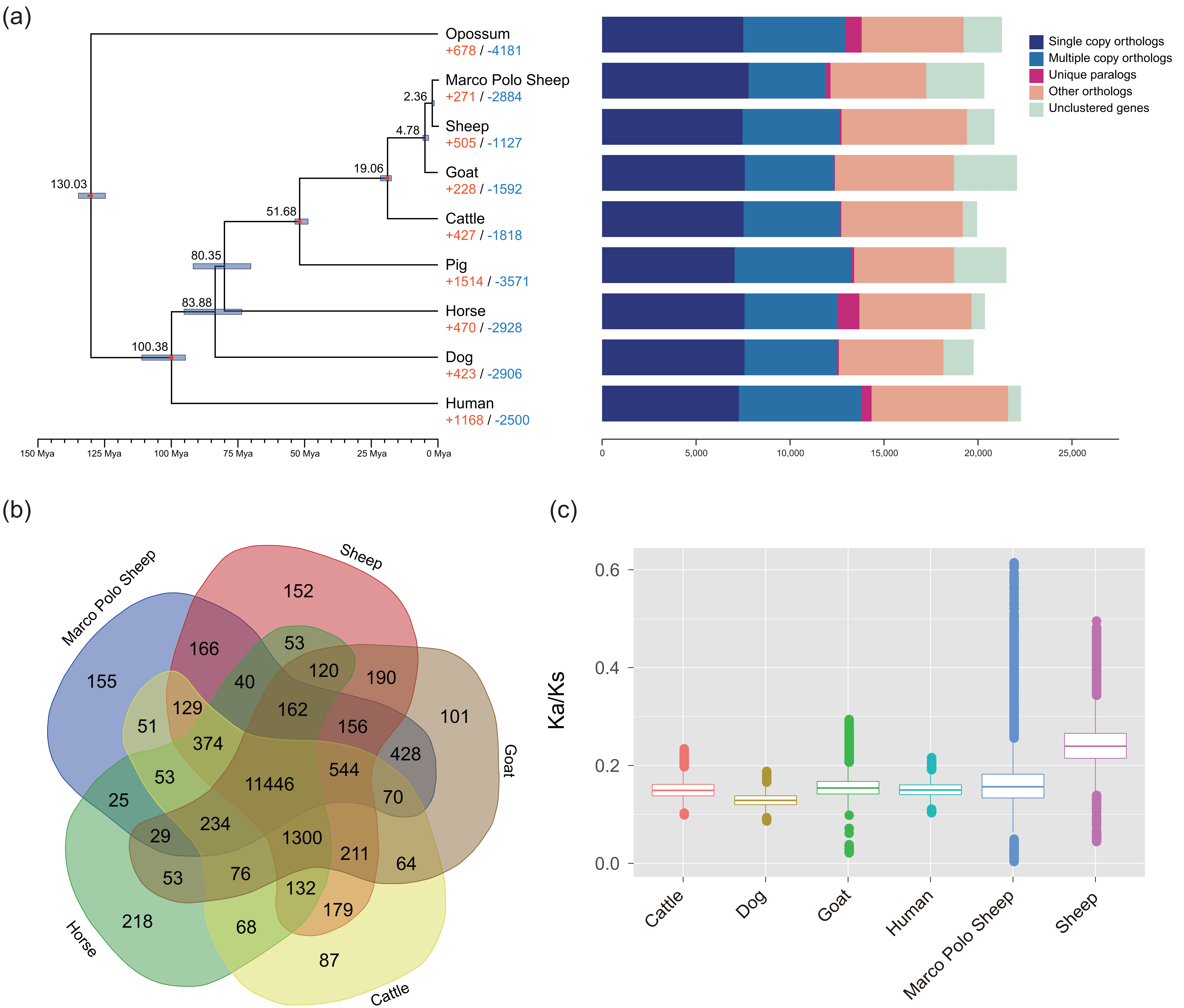

Click here to access/download
Supplementary Material
Macro polo sheep-Supplementary files.pdf

Response letter

Click here to access/download
Supplementary Material
Response_to_reviewer_comments.docx

Dear Editor,

Thank you very much for returning our manuscript GIGA-D-17-00160 entitled "The genome of the Marco Polo Sheep (*Ovis ammon polii*)", along with the helpful comments from the communicating editor and the referees.

We highly appreciated the constructive and thoughtful comments from the reviewers. Their suggestions have helped to improve the manuscript greatly. We have carefully revised the manuscript according to their suggestions and comments, and responded point by point to their comments as itemized below (our responses are marked with the **BOLD** type). We have asked a professional institution to polish English of this present version. We have renamed genes (common name was included now) and modified three files in GigaDB (Marco.Polo.Sheep.gene.gff3.gz, Marco.Polo.Sheep.gene.cds.fa.gz and Marco.Polo.Sheep.gene.pep.fa.gz). In addition, a detailed description of each author's role in this work had been added.

We submit here our revised manuscript and hope that the revised manuscript is more suitable for the publication in ***GigaScience***. If you have any questions, please do not hesitate to contact the corresponding author at any time.

Thank you again for your time and efforts in handling our manuscript.

Best wishes,

Kun Wang

Center for Ecological and Environmental Sciences, Northwestern Polytechnical University, Xi'an 710072, China
