# Supplementary material for: Draft genome of the Marco Polo Sheep (Ovis ammon polii)
Source: Gigascience. 2017 Nov 1;6(12):1–7. doi: 10.1093/gigascience/gix106 (PMC5740985; doi:10.1093/gigascience/gix106)
Supplement: Supplementary figures and tables [file gix106_supp.pdf]

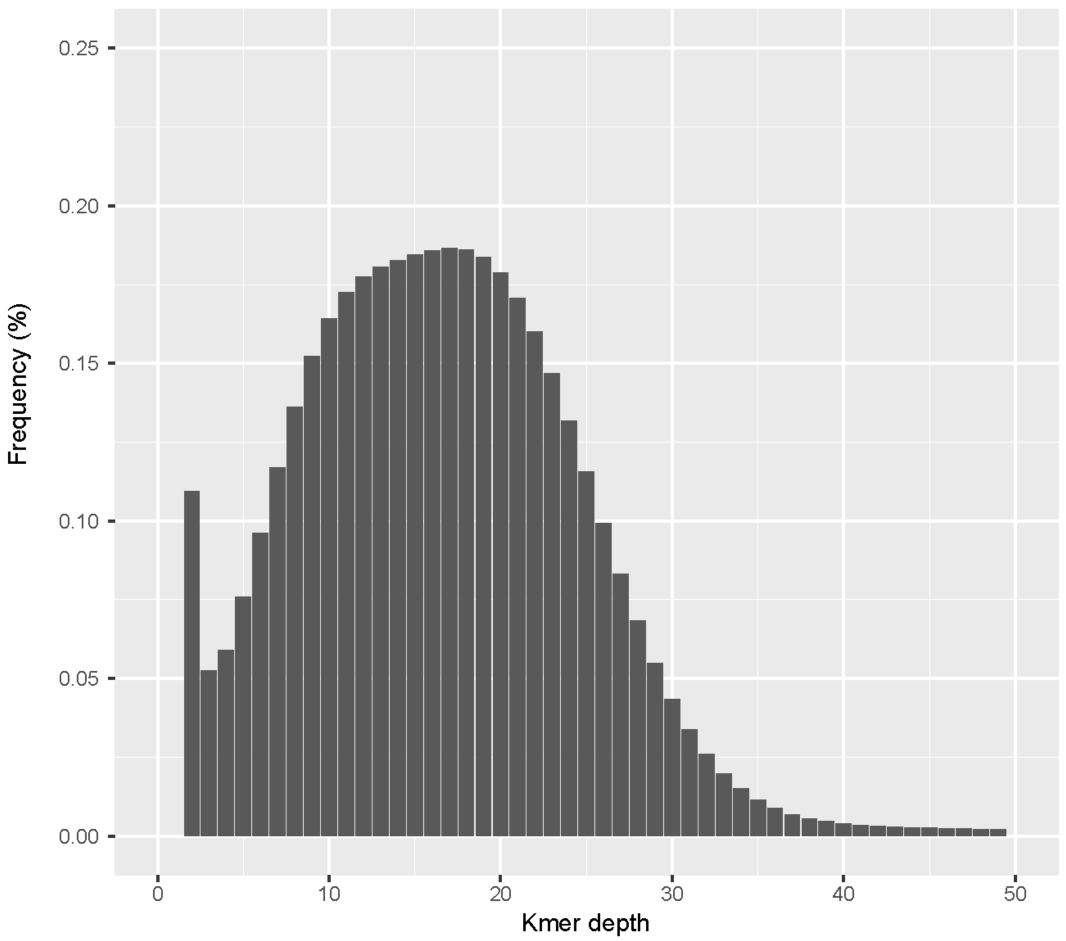

Figure S1. 21-mer-based analysis carried out to estimate the size of the Marco Polo Sheep genome.

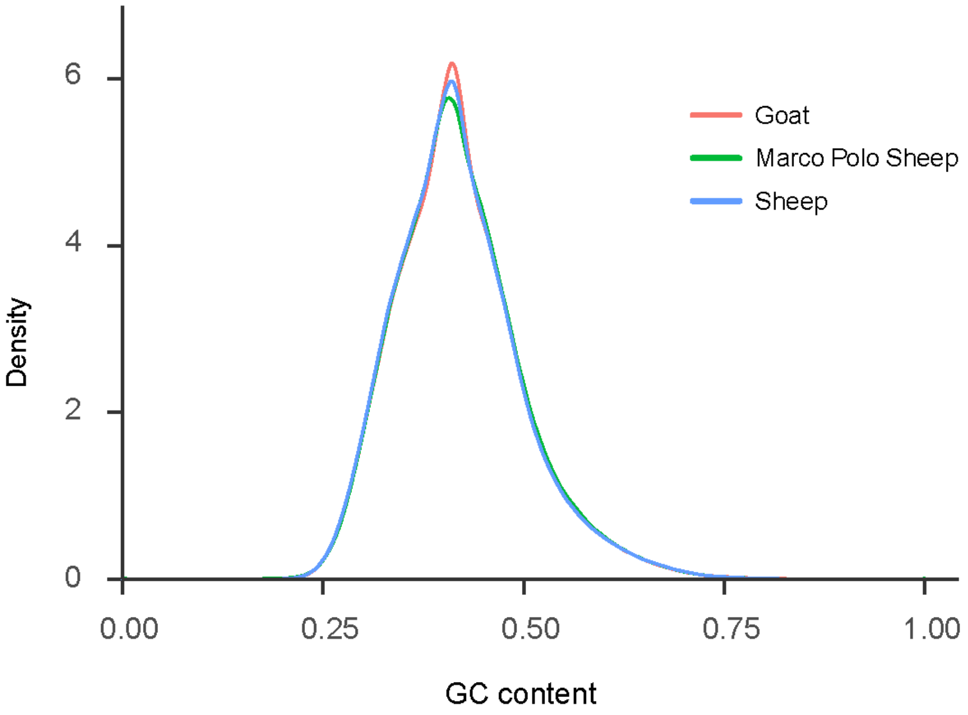

Figure S2. **GC content distribution for the genomes of Marco Polo Sheep, goat (ARS1) and sheep (Oar_v3.1).** The GC content were established by 500 bp non-overlapping sliding windows.

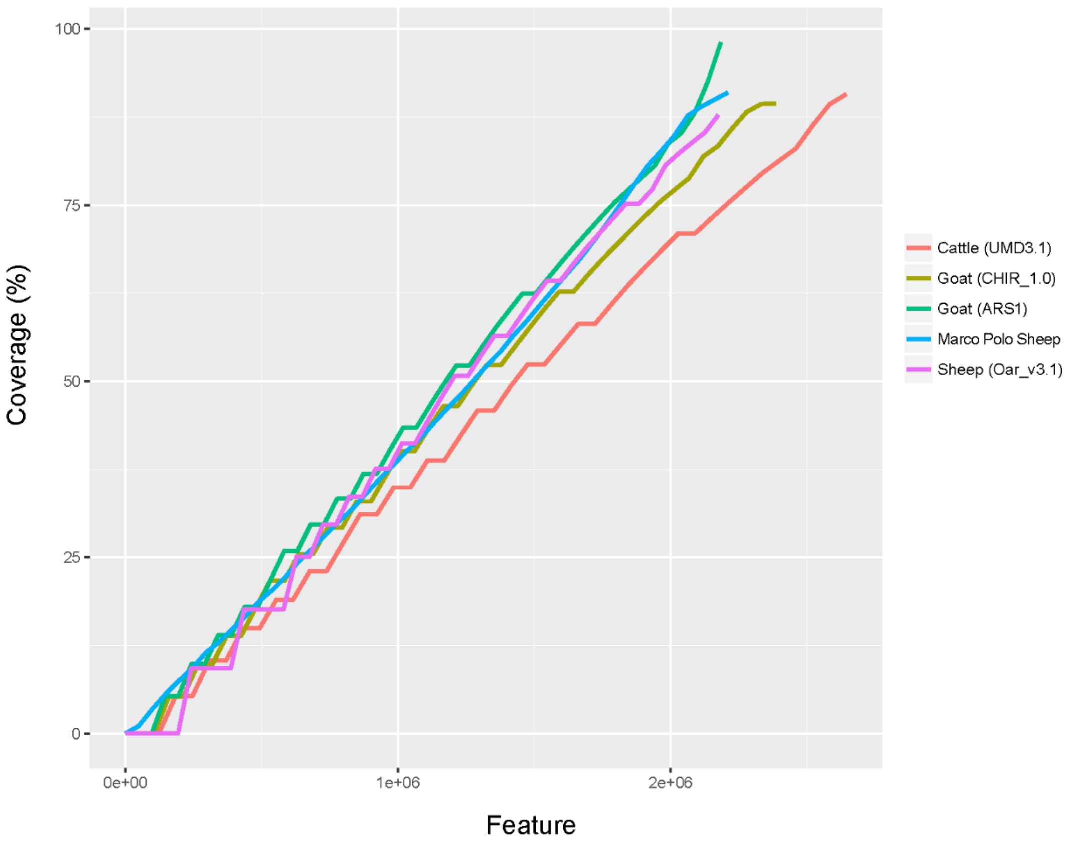

Figure S3. **FRCurve of five genome assemblies.** The FRCurve were calculated by the software FRC_align (https://github.com/vezzi/FRC_align)

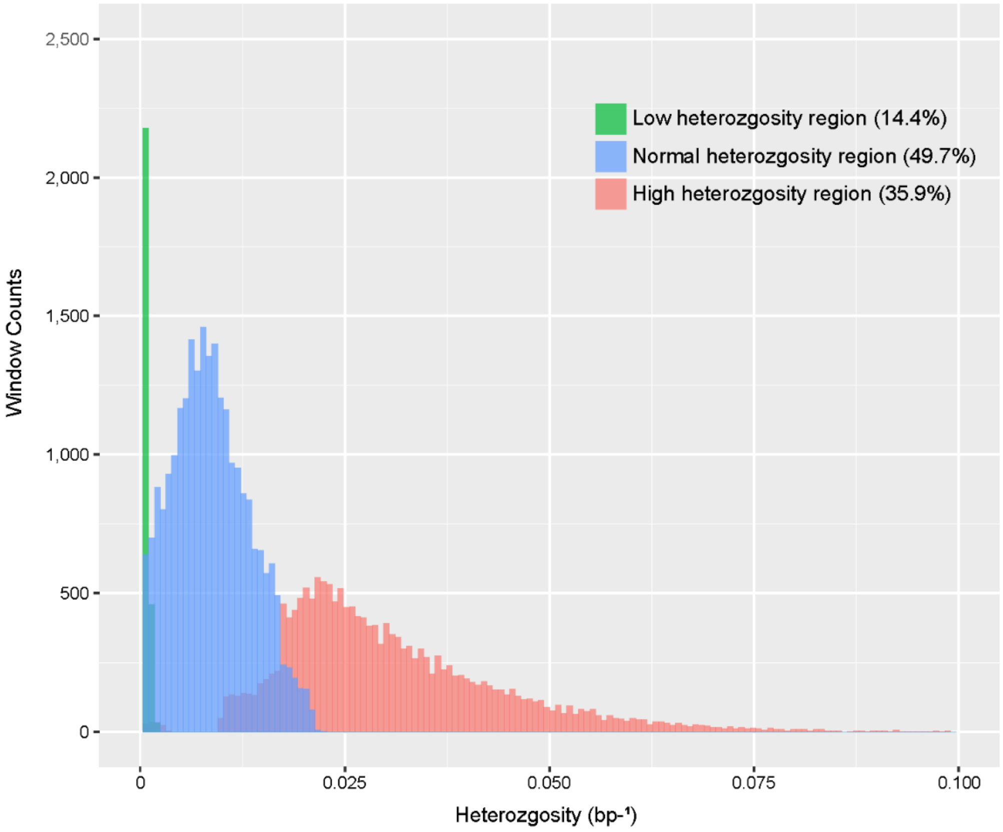

Figure S4. The distribution of observed heterozgosity stats within Marco Polo Sheep genome. The stats of low, normal and high heterozgosity regions were identified by depmixS4 package and the proportion of each stat were mark within the brackets.

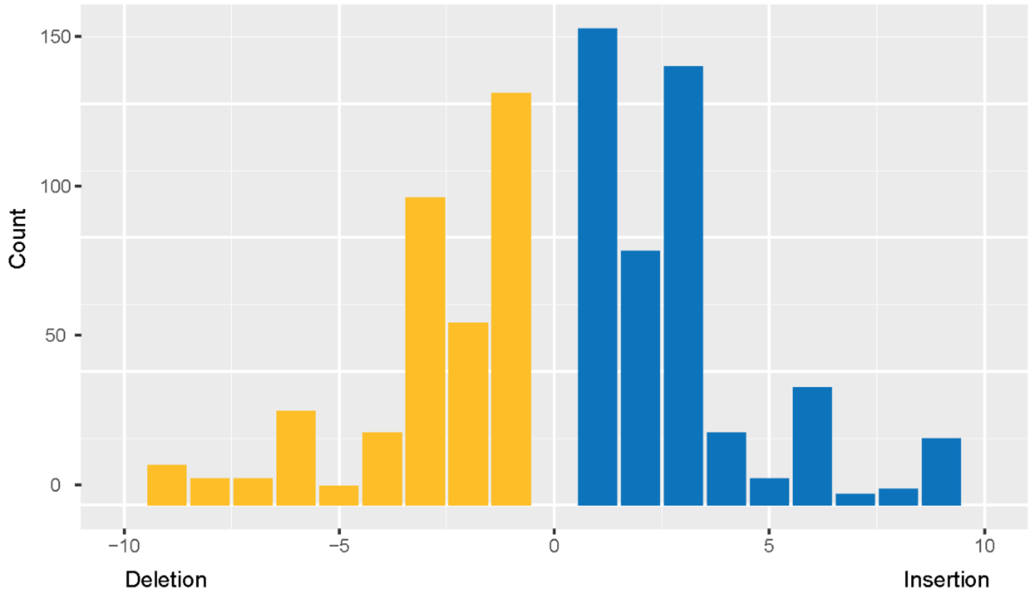

Figure S5. Counts of InDels in coding regions, showing an enrichment of multiples of three bases.

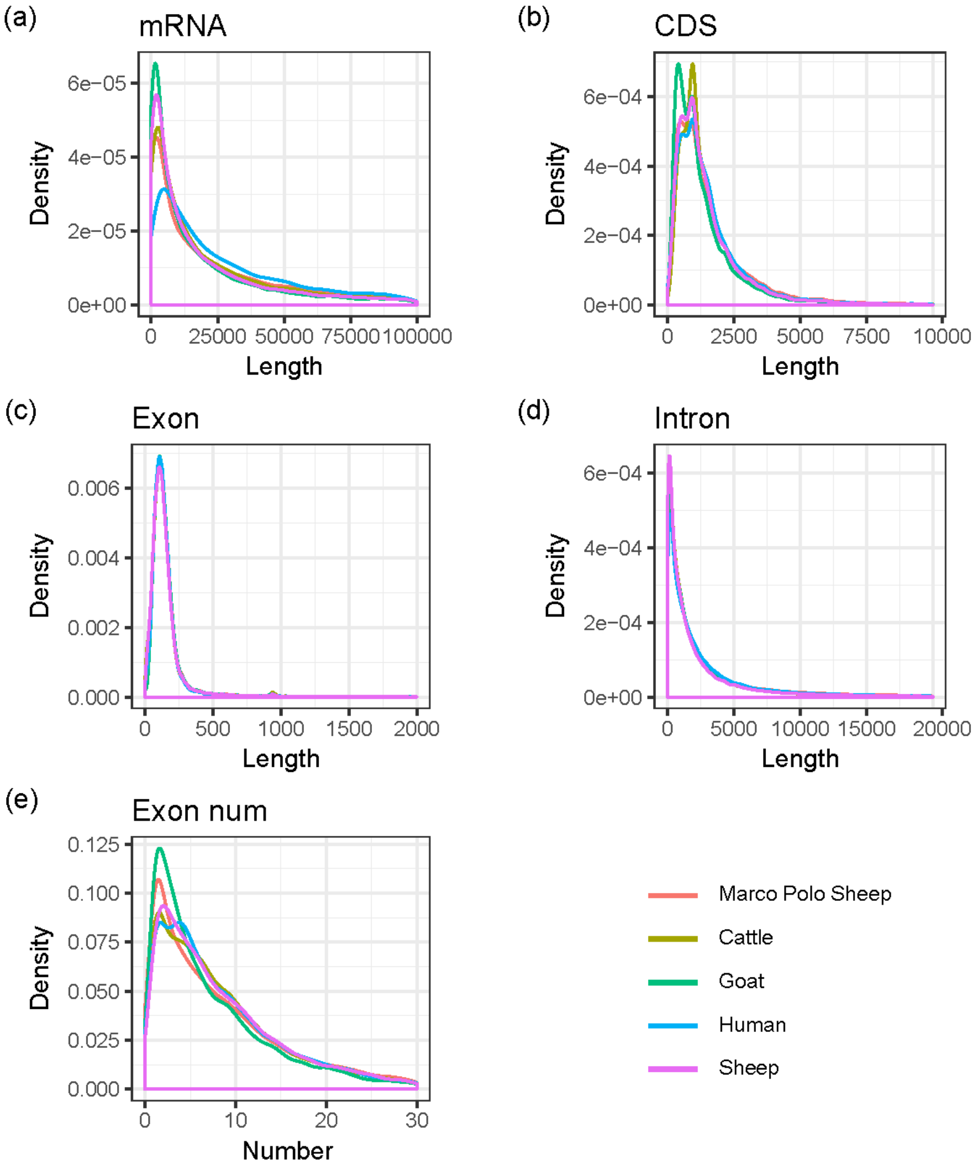

Figure S6. Comparison of gene structure characteristics with those of other mammals. (a) mRNA length, (b) CDS length, (c) Exon length, (d) Intron length, and (e) Exon number per gene between Marco Polo Sheep, Cattle (UMD3.1), Goat (CHIR_1.0), Human (GRCh38) and Sheep (Oar_v3.1). The *x*-axis represents length or number and the *y*-axis represents the density of genes.

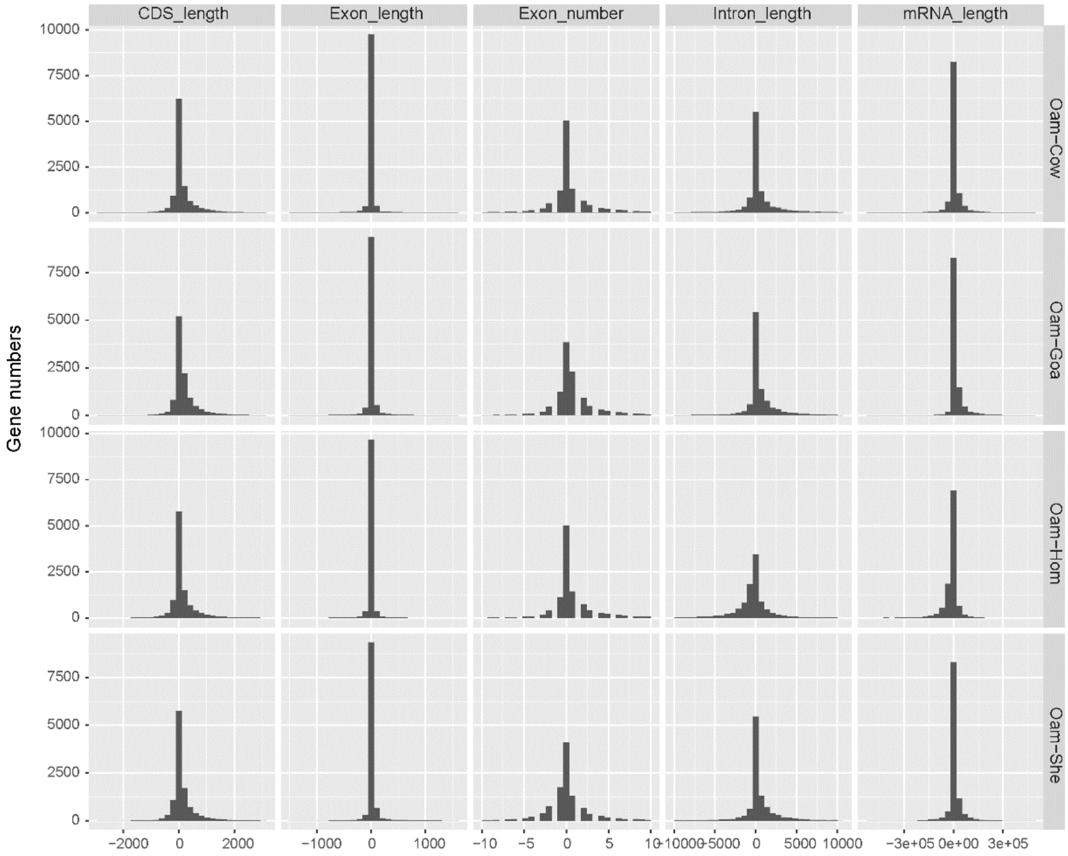

Figure S7. Comparison of gene structure characteristics of the 1:1 orthologs in the five mammals. The *x*-axis represents length or number in Marco Polo Sheep genome minus the corresponding values in other mammals. The aliases or abbreviation: Oam (Marco Polo Sheep), Cow (Cattle, UMD3.1), Goa (Goat, CHIR_1.0), Hom (Human, GRCh38) and She (Sheep, Oar_v3.1).

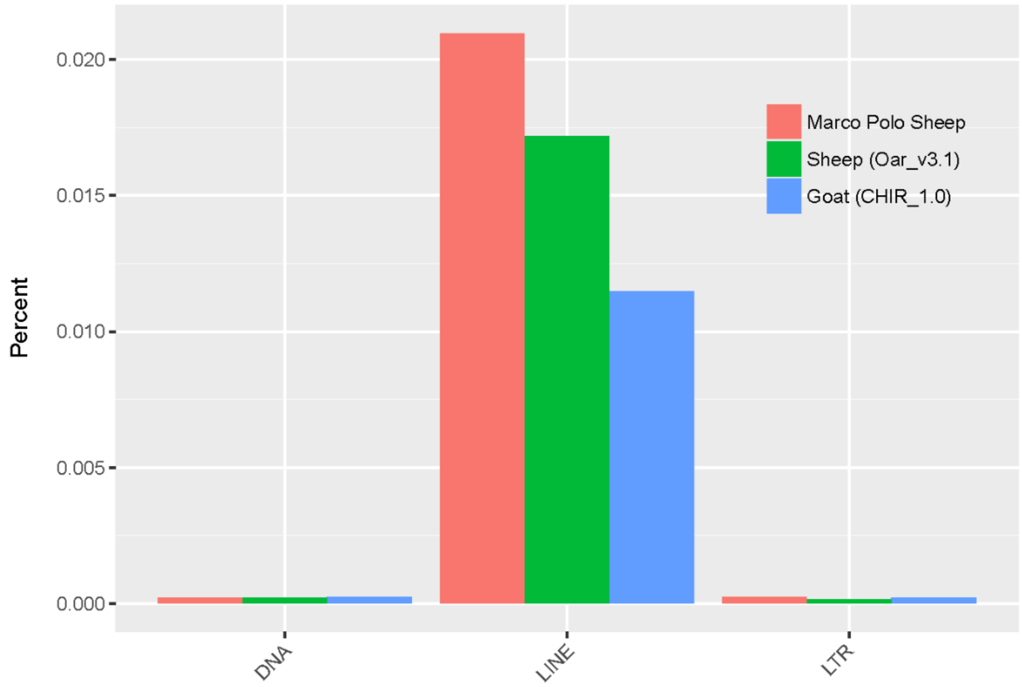

Figure S8. Comparison of the repeat content in the intron regions among Marco Polo Sheep, Sheep (Oar_v3.1) and Goat (CHIR_1.0).

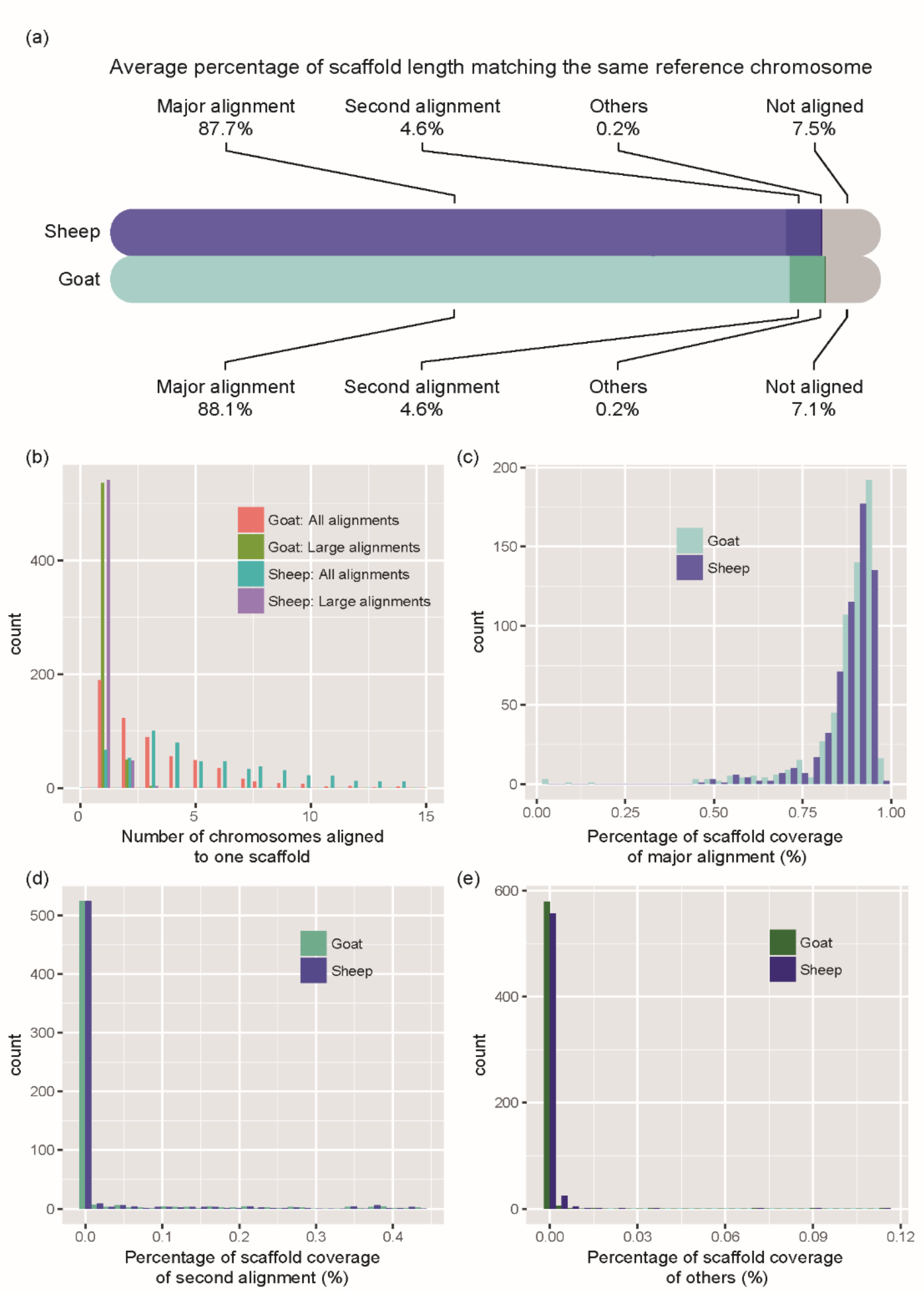

Figure S9. Summary of the number of chromosomes to which a given scaffold of the Marco Polo Sheep genome could be aligned. (a) The length of alignments from the same reference chromosome (Goat, ARS1 and Sheep, Oar_v3.1) were summed. The alignments (sum for one reference chromosome) with the largest percent of the length of a Marco Polo Sheep scaffold was named ‘Major alignment’. The alignments (sum of one chromosome of reference) with second largest percent of the length was named as ‘Second alignment’. The other alignments (from the other chromosomes) was named as ‘Others’. (b) The alignment (from one chromosome) with a percent larger than 5% of a scaffold (not ‘Major alignment’) was named ‘Large alignment’. (c) Histogram of percent of ‘Major alignment’. (d) Histogram of percent of ‘Second alignment’. (e) Histogram of percent of ‘Others’.

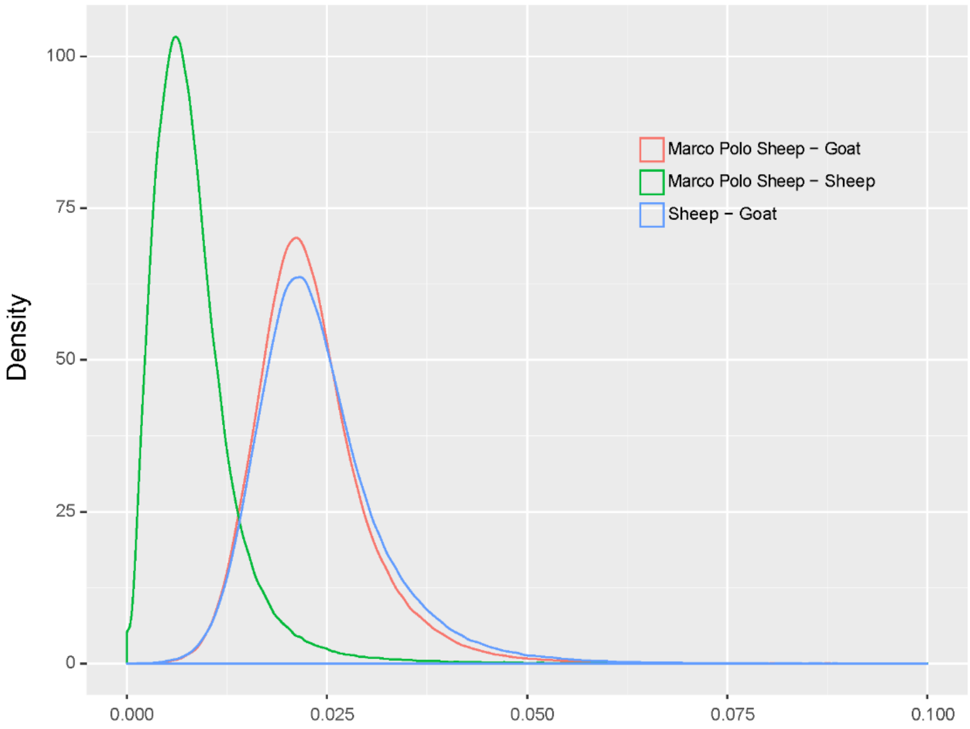

Figure S10. Divergence between Marco Polo Sheep, sheep (Oar_v3.1) and goat (ARS1).

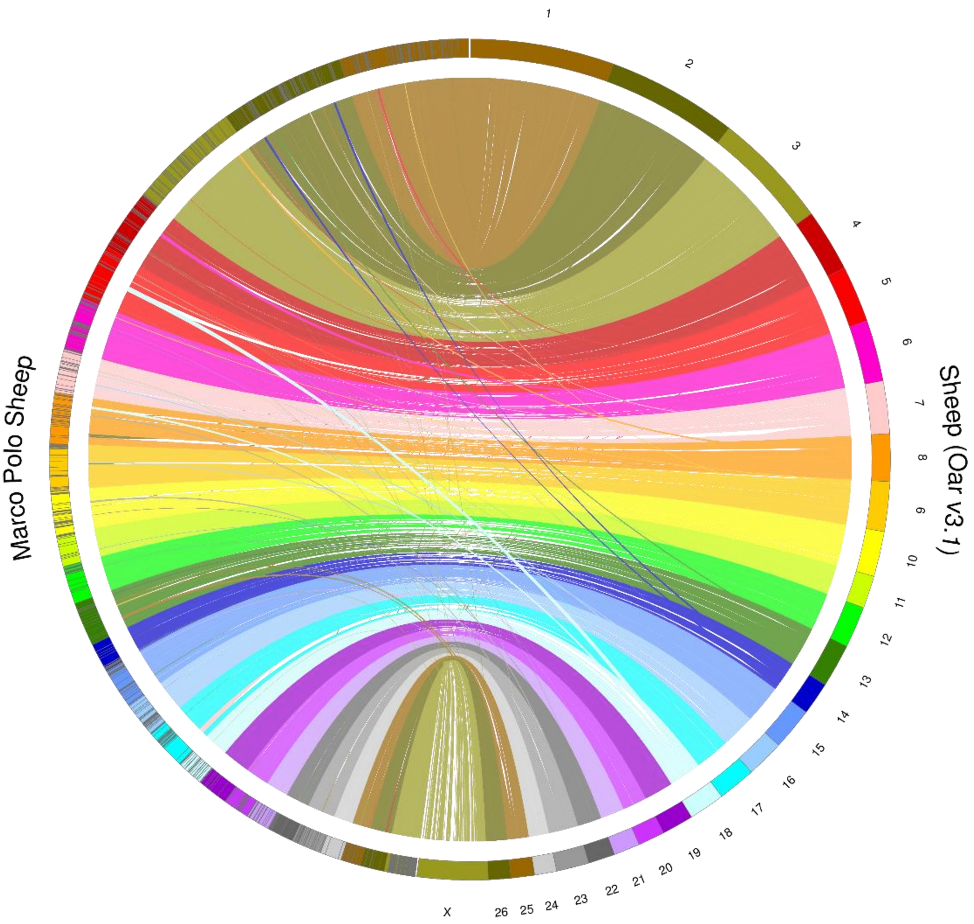

Figure S11. Synteny relationship between Marco Polo Sheep and sheep (Oar_v3.1).

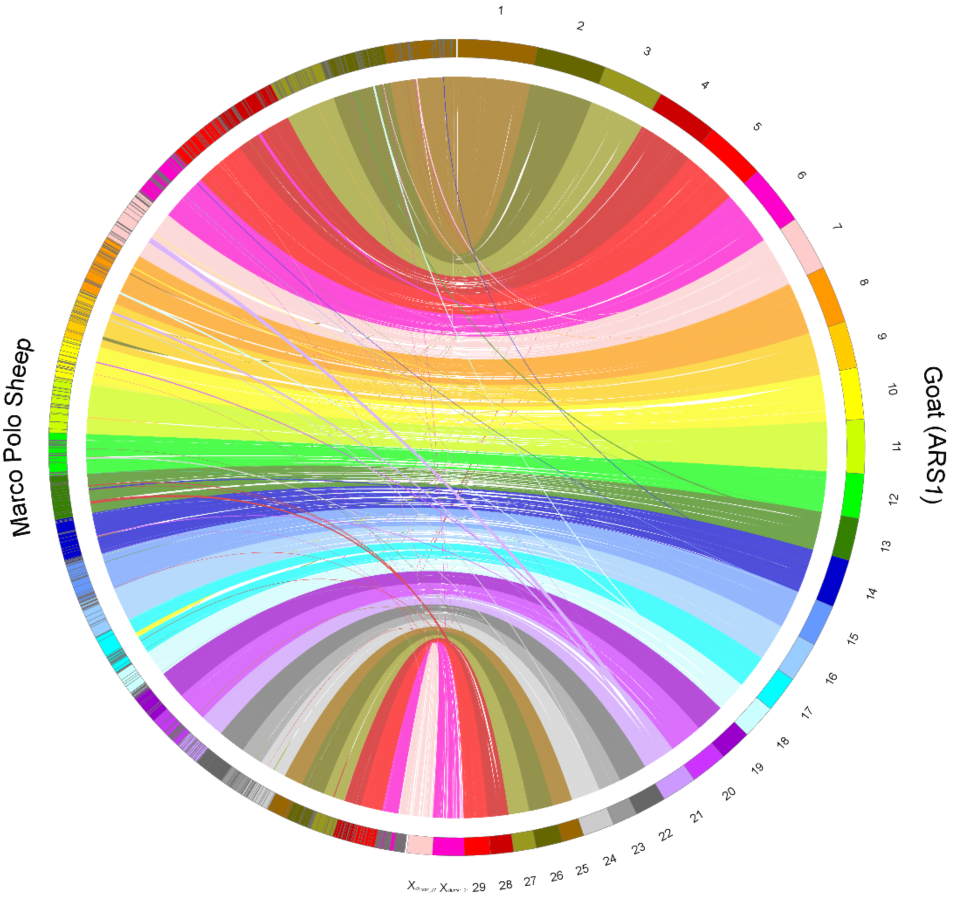

Figure S12. Synteny relationship between Marco Polo Sheep and goat (ARS1). The goat assembly contained two fragment of X chromosome: X cluster 21 and X cluster 27.

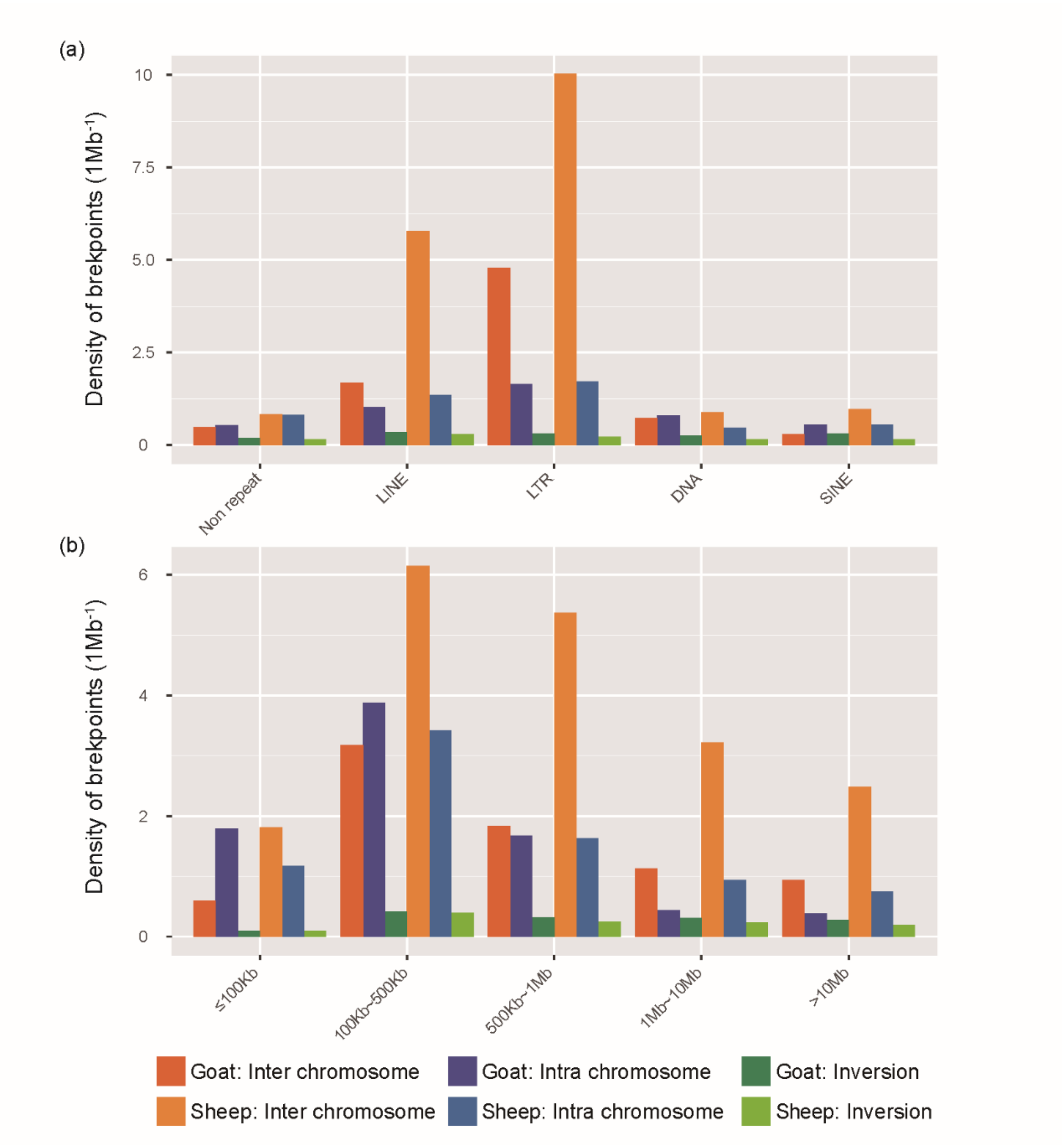

Figure S13. Density of breakpoints (number per million bases) in different regions of the genome. (a) The breakpoints were counted in non-repeat regions and different types of repetitive regions. (b) The breakpoints were counted in scaffolds with different lengths. The genome version: Goat (ARS1) and Sheep (Oar_v3.1).

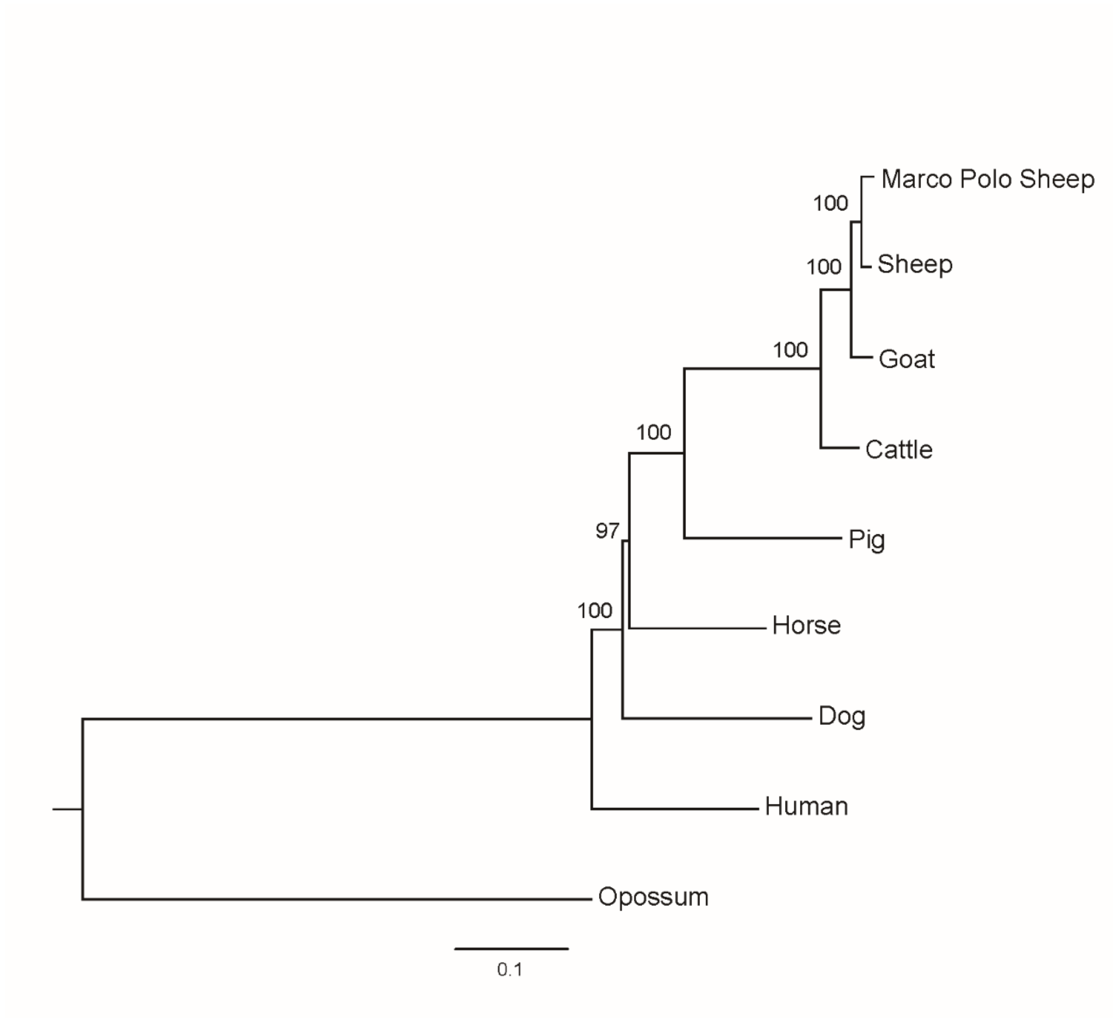

Figure S14. Phylogeny relationships between Marco Polo Sheep and other mammals.

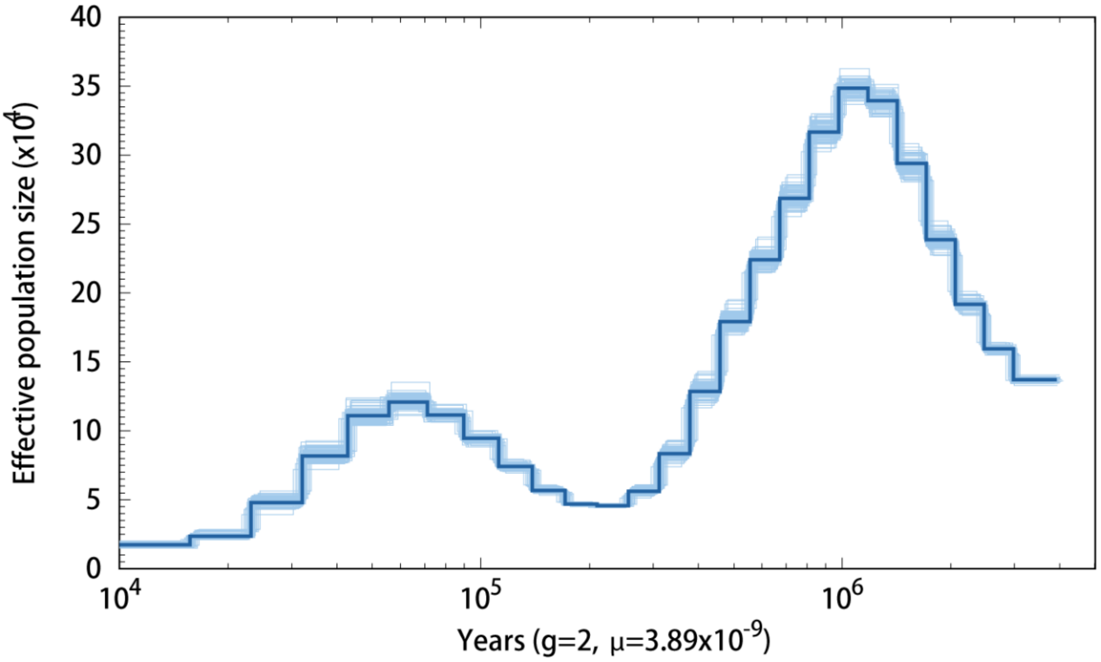

Figure S15. Demographic history of Marco Polo Sheep.

Table S1. Summary of sequenced reads.

Library Insert Size	Raw Reads				Qualified Reads[1]				SRA number
	**Total Data (Gb)**	**Read Length (bp)**	**Sequence Coverage[2] (X)**	**Physical Coverage[2] (X)**	**Total Data (Gb)**	**Read Length (bp)**	**Sequence Coverage[2] (X)**	**Physical Coverage[2] (X)**	
400	124.25	149.93	41.42	55.25	103.22	136.58	34.41	50.38	SRR5753923
500	87.47	149.93	29.16	48.62	74.25	135.45	24.75	45.68	SRR5753922
600	103.83	149.93	34.61	69.25	88.39	134.66	29.46	65.63	SRR5753921
700	112.18	150.00	37.39	87.25	95.55	135.69	31.85	82.16	SRR5753920
800	76.34	150.00	25.45	67.86	63.88	134.08	21.29	63.52	SRR5753919
4000	72.59	150.00	24.20	322.63	32.03	140.00	10.68	152.53	SRR5753918
8000	72.04	150.00	24.01	640.33	39.38	140.00	13.13	375.09	SRR5753917
10000	105.62	150.00	35.21	1,173.53	47.10	140.00	15.70	560.76	SRR5753915
12000	59.10	150.00	19.70	788.02	25.95	140.00	8.65	370.73	SRR5753914
15000	209.01	150.00	69.67	3,483.56	54.98	140.00	18.33	981.79	SRR5753913, SRR5753912
**Total**	**1,022.43**	**149.98**	**340.81**	**6,736.30**	**624.74**	**136.84**	**208.25**	**2,748.28**	

[1]Qualified reads were generated by filtering the low quality reads, base-calling duplicate and adapter contamination from the raw reads. For the short insert libraries, we further filtered employed the kmer-based correction.

[2]Coverage was calculated under the assumption of a genome size of 3 Gb for Marco Polo Sheep. Sequence coverage refers to the total length of generated reads, and physical coverage refers to the total cloned DNA used for the paired reads.

Table S2. Estimation of genome size based on 21-mer statistics.

K-mer Value	K-mer Number	K-mer Depth	Genome Size (Gb)	Used Bases (Gb)	Used Reads	Depth (X)
21	52,413,427,492	17	3.083	65.690	663,523,670	21

Table S3. Statistics for the final assemblies of the Marco Polo Sheep genome.

	Contig		Scaffold	
	**Size (bp)**	**Number**	**Size (bp)**	**Number**
**N90**	2,725	110,374	3,655	6,774
**N80**	10,129	65,669	810,793	462
**N70**	16,585	45,835	2,492,680	276
**N60**	23,220	32,665	3,799,082	190
**N50**	30,772	22,965	5,492,388	131
**Longest**	479,502		31,456,455	
**Total Size**	2,593,195,672		2,711,208,465	
**Total Number (>100 bp)**	726,493		620,343	
**Total Number (>2 kb)**	132,912		33,717	

Table S4. Numbers of reads mapped to the assembled Marco Polo Sheep genome.

**Library Insert Size (bp)**	**Total pairs (PE) reads**	**Pair end mapped reads**	**Pair end mapped ratio (%)**	**Single end mapped reads**	**Single end mapped ratio (%)**	**Total mapped reads**	**Total mapped ratio (%)**
400	755,764,962	752,581,356	99.58	883,511	0.12	753,464,867	99.70
500	548,188,304	545,693,218	99.54	990,943	0.18	546,684,161	99.72
600	656,344,938	652,775,350	99.46	1,588,826	0.24	654,364,176	99.70
700	704,223,564	700,335,458	99.45	1,512,462	0.21	701,847,920	99.66
800	476,414,830	471,398,348	98.95	2,024,164	0.42	473,422,512	99.37
4,000	228,796,784	226,404,764	98.95	1,056,311	0.46	227,461,075	99.42
8,000	281,319,370	279,749,360	99.44	684,604	0.24	280,433,964	99.69
10,000	336,456,790	330,052,302	98.10	2,733,310	0.81	332,785,612	98.91
12,000	185,362,556	183,733,284	99.12	722,478	0.39	184,455,762	99.51
15,000	392,714,212	387,657,160	98.71	2,160,731	0.55	389,817,891	99.26
**Total**	**4,565,586,310**	**4,530,380,600**	**99.23**	**14,357,340**	**0.31**	**4,544,737,940**	**99.54**

Table S5. Summary of CEGMA analysis results.

Species		#Proteins [1]	Completeness (%) [2]	#Total [3]	Average [4]	Ortho (%) [5]
Marco polo sheep	Complete	169	68.15	344	2.04	52.07
	Group 1	40	60.61	65	1.62	40
	Group 2	37	66.07	62	1.68	48.65
	Group 3	41	67.21	86	2.1	56.1
	Group 4	51	78.46	131	2.57	60.78
	Partial	232	93.55	615	2.65	72.84
	Group 1	60	90.91	131	2.18	65
	Group 2	52	92.86	124	2.38	69.23
	Group 3	59	96.72	161	2.73	74.58
	Group 4	61	93.85	199	3.26	81.97
Sheep (Oar v3.1)	Complete	156	62.9	263	1.69	40.38
	Group 1	34	51.52	50	1.47	29.41
	Group 2	36	64.29	54	1.5	33.33
	Group 3	39	63.93	63	1.62	38.46
	Group 4	47	72.31	96	2.04	55.32
	Partial	228	91.94	537	2.36	68.86
	Group 1	55	83.33	112	2.04	60
	Group 2	52	92.86	105	2.02	53.85
	Group 3	61	100	141	2.31	72.13
	Group 4	60	92.31	179	2.98	86.67
Goat (ARS1)	Complete	162	65.32	283	1.75	40.12
	Group 1	37	56.06	57	1.54	35.14
	Group 2	38	67.86	57	1.5	31.58
	Group 3	38	62.3	65	1.71	36.84
	Group 4	49	75.38	104	2.12	53.06
	Partial	233	93.95	543	2.33	67.81
	Group 1	60	90.91	121	2.02	60
	Group 2	52	92.86	101	1.94	51.92
	Group 3	60	98.36	144	2.4	75
	Group 4	61	93.85	177	2.9	81.97
Goat (CHIR_1.0)	Complete	144	58.06	246	1.71	39.58
	Group 1	35	53.03	48	1.37	25.71
	Group 2	31	55.36	44	1.42	29.03
	Group 3	34	55.74	59	1.74	41.18
	Group 4	44	67.69	95	2.16	56.82
	Partial	232	93.55	526	2.27	66.81
	Group 1	58	87.88	113	1.95	60.34
	Group 2	52	92.86	102	1.96	53.85
	Group 3	61	100	139	2.28	67.21
	Group 4	61	93.85	172	2.82	83.61

[1]number of 248 ultra-conserved core eukaryotic genes (CEGs) present in genome;

[2]percentage of 248 ultra-conserved CEGs present;

[3]total number of CEGs present including putative orthologs;

[4]average number of orthologs per CEG;

[5]percentage of detected CEGS having more than 1 ortholog.

Table S6. Summary of BUSCO analysis results obtained by counting matches to 4104 single-copy orthologs (mammalia_odb9).

BUSCO mode	Species	Complete one-to-one match to ortholog	Complete match of multi gene copies to ortholog	Fragmented match to ortholog	Total number of matches to ortholog	No match to ortholog
Genome	Marco polo sheep	3749 (91.3%)	51 (1.2%)	139 (3.4%)	3939 (95.9%)	165 (4.1%)
	Sheep (Oar_v3.1)	3776 (92.0%)	46 (1.1%)	142 (3.5%)	3964 (96.6%)	140 (3.4%)
	Goat (ARS1)	3809 (92.8%)	41 (1.0%)	124 (3.0%)	3974 (96.8%)	130 (3.2%)
	Goat (CHIR_1.0)	3769 (91.8%)	35 (0.9%)	159 (3.9%)	3963 (96.6%)	141 (3.4%)
OGS	Marco polo sheep	3746 (91.3%)	43 (1.0%)	159 (3.9%)	3948 (96.2%)	156 (3.8%)
	Sheep (Oar_v3.1)	4006 (97.6%)	37 (0.9%)	40 (1.0%)	4083 (99.5%)	21 (0.5%)
	Goat (ARS1)	3978 (96.9%)	38 (0.9%)	30 (0.7%)	4046 (98.5%)	58 (1.5%)
	Goat (CHIR_1.0)	3940 (96.0%)	28 (0.7%)	88 (2.1%)	4056 (98.8%)	48 (1.2%)

Table S7. The distribution of SNVs in the Marco Polo Sheep genome.

Location	Count
Inter-genetic	2,368,356
Intron	1,165,301
synonymous variant	17,410
missense variant	
missense	14,487
stop gain	263
stop lost	43
start lost	61
**Total**	**3,565,943**

Table S8. Genes located in the low heterozygosity regions.

Marco Polo Sheep gene id	Gene symbol	Gene name
scaffold1009_G001	NA	NA
scaffold1009_G002	NA	NA
scaffold1030_G001_PRAME	PRAME	preferentially expressed antigen in melanoma
scaffold1031_G001	NA	NA
scaffold1033_G001_ZNF449	ZNF449	zinc finger protein 449
scaffold1033_G002_ZNF75D	ZNF75D	zinc finger protein 75D
scaffold1035_G001_FMR1	FMR1	fragile X mental retardation 1
scaffold1048_G001_TRIM69	TRIM69	tripartite motif containing 69
scaffold1049_G001_FUNDC1	FUNDC1	FUN14 domain containing 1
scaffold1067_G001_CITED1	CITED1	Cbp/p300 interacting transactivator with Glu/Asp rich carboxy-terminal domain 1
scaffold1077_G001_DUSP21	DUSP21	dual specificity phosphatase 21
scaffold1079_G001_MKRN1	MKRN1	Makorin Ring Finger Protein 1
scaffold1083_G001	NA	NA
scaffold1086_G001_MSN	MSN	moesin
scaffold1089_G001_FGF16	FGF16	fibroblast growth factor 16
scaffold1102_G001_EDA	EDA	ectodysplasin A
scaffold1102_G002_AWAT2	AWAT2	acyl-CoA wax alcohol acyltransferase 2
scaffold1105_G001_ZC4H2	ZC4H2	zinc finger C4H2-type containing
scaffold1110_G001_CXorf36	CXorf36	chromosome X open reading frame 36
scaffold1123_G001_LAMP2	LAMP2	lysosomal associated membrane protein 2
scaffold1138_G001	NA	NA
scaffold1159_G001	NA	NA
scaffold1160_G001	NA	NA
scaffold1169_G001	NA	NA
scaffold1169_G002_TEX13A	TEX13A	Testis Expressed 13A
scaffold1177_G001_FAM120C	FAM120C	Family With Sequence Similarity 120C
scaffold1181_G001	NA	NA
scaffold1181_G002_VBP1	VBP1	VHL binding protein 1
scaffold1182_G001_MAGEA10	MAGEA10	MAGE Family Member A10
scaffold1187_G001	NA	NA
scaffold1192_G001	NA	NA
scaffold1198_G001	NA	NA
scaffold1201_G001	NA	NA
scaffold1223_G001_PGK1	PGK1	phosphoglycerate kinase 1
scaffold1223_G002	NA	NA
scaffold1225_G001_AR	AR	androgen receptor
scaffold1228_G001_TRPC5OS	TRPC5OS	
scaffold1239_G001_DDX53	DDX53	DEAD-box helicase 53
scaffold124_G001_ZNF596	ZNF596	Zinc Finger Protein 596
scaffold1248_G001_TMEM47	TMEM47	Transmembrane Protein 47
scaffold1251_G001_JADE3	JADE3	jade family PHD finger 3

scaffold1269_G001	NA	NA
scaffold1274_G001	NA	NA
scaffold1285_G001_MOSPD2	MOSPD2	motile sperm domain containing 2
scaffold1286_G001_PDZD11	PDZD11	PDZ domain containing 11
scaffold1286_G002_ARR3	ARR3	arrestin 3
scaffold1286_G003_P2RY4	P2RY4	Pyrimidinergic Receptor P2Y4
scaffold1294_G001_SHROOM4	SHROOM4	shroom family member 4
scaffold1302_G001	NA	NA
scaffold1302_G002_PFKFB1	PFKFB1	6-phosphofructo-2-kinase/fructose-2,6-biphosphatase 1
scaffold1303_G001_USP26	USP26	ubiquitin specific peptidase 26
scaffold1312_G001_IRS4	IRS4	insulin receptor substrate 4
scaffold1317_G001_APEX2	APEX2	apurinic/apyrimidinic endodeoxyribonuclease 2
scaffold1317_G002_ALAS2	ALAS2	5'-aminolevulinate synthase 2
scaffold1320_G001	NA	NA
scaffold1336_G001	NA	NA
scaffold1347_G001	NA	NA
scaffold1350_G001_PHF6	PHF6	PHD finger protein 6
scaffold1361_G001	NA	NA
scaffold1370_G001	NA	NA
scaffold1370_G002	NA	NA
scaffold1374_G001	NA	NA
scaffold1385_G001_RAB9B	RAB9B	RAB9B, member RAS oncogene family
scaffold1389_G001	NA	NA
scaffold1420_G001_IL13RA1	IL13RA1	interleukin 13 receptor subunit alpha 1
scaffold1433_G001_LPAR4	LPAR4	lysophosphatidic acid receptor 4
scaffold1467_G001_RTL4	RTL4	Retrotransposon Gag Like 4
scaffold1472_G001	NA	NA
scaffold1475_G001	NA	NA
scaffold1482_G001	NA	NA
scaffold1483_G001_DGAT2L6	DGAT2L6	diacylglycerol O-acyltransferase 2 like 6
scaffold1511_G001	NA	NA
scaffold1511_G002	NA	NA
scaffold1511_G003_PPP4R3CP	PPP4R3CP	protein phosphatase 4 regulatory subunit 3C, pseudogene
scaffold1517_G001	NA	NA
scaffold153_G001_RAB24	RAB24	RAB24, member RAS oncogene family
scaffold1546_G001_TBX22	TBX22	T-box 22
scaffold1564_G001_BMP15	BMP15	bone morphogenetic protein 15 precursor
scaffold1578_G001	NA	NA
scaffold1591_G001_CDX4	CDX4	caudal type homeobox 4
scaffold1597_G001_ALG13	ALG13	ALG13, UDP-N-acetylglucosaminyltransferase subunit
scaffold1600_G001	NA	NA

scaffold1602_G001_TEX13D	TEX13D	TEX13 Family Member D
scaffold1609_G001	NA	NA
scaffold1635_G001_POU3F4	POU3F4	POU class 3 homeobox 4
scaffold1643_G001_LDOC1	LDOC1	LDOC1, Regulator Of NFKB Signaling
scaffold1648_G001	NA	NA
scaffold1652_G001	NA	NA
scaffold1653_G001	NA	NA
scaffold1704_G001_P2RY10	P2RY10	purinergic receptor P2Y10
scaffold1804_G001	NA	NA
scaffold1851_G001	NA	NA
scaffold1865_G001_BEX5	BEX5	brain expressed X-linked 5
scaffold1889_G001	NA	NA
scaffold1925_G001_FOXR2	FOXR2	forkhead box R2
scaffold194_G001_DLA-DQB1	DLA-DQB1	major histocompatibility complex, class II, DQ beta 1
scaffold1948_G001	NA	NA
scaffold2_G001	NA	NA
scaffold2_G002_ZNHIT1	ZNHIT1	zinc finger HIT-type containing 1
scaffold2_G003_CLDN15	CLDN15	claudin 15
scaffold2_G004_FIS1	FIS1	fission, mitochondrial 1
scaffold2004_G001	NA	NA
scaffold2013_G001	NA	NA
scaffold2016_G001	NA	NA
scaffold2034_G001	NA	NA
scaffold2039_G001	NA	NA
scaffold2061_G001	NA	NA
scaffold2202_G001	NA	NA
scaffold2214_G001	NA	NA
scaffold2253_G001	NA	NA
scaffold2293_G001	NA	NA
scaffold23_G001_SPOUT1	SPOUT1	SPOUT domain containing methyltransferase 1
scaffold23_G002_TBC1D13	TBC1D13	TBC1 domain family member 13
scaffold25_G001_CHAMP1	CHAMP1	chromosome alignment maintaining phosphoprotein 1
scaffold267_G001_ERBIN	ERBIN	erbb2 interacting protein
scaffold315_G001	NA	NA
scaffold350_G001_CCNB3	CCNB3	cyclin B3
scaffold373_G001	NA	NA
scaffold412_G001_ZNF275	ZNF275	zinc finger protein 275
scaffold418_G001	NA	NA
scaffold436_G001_GPR50	GPR50	melatonin-related receptor
scaffold444_G001_TMEM192	TMEM192	transmembrane protein 192
scaffold546_G001_EFNB1	EFNB1	ephrin B1
scaffold564_G001	NA	NA

scaffold576_G001	NA	NA
scaffold576_G002	NA	NA
scaffold603_G001_CXCR3	CXCR3	C-X-C motif chemokine receptor 3
scaffold621_G001_MAP7D2	MAP7D2	MAP7 domain containing 2
scaffold628_G001_PHF24	PHF24	PHD finger protein 24
scaffold639_G001_CENPI	CENPI	centromere protein I
scaffold661_G001	NA	NA
scaffold691_G001_DRP2	DRP2	dystrophin related protein 2
scaffold727_G001_OR9G1	OR9G1	olfactory receptor family 9 subfamily G member 1
scaffold727_G002	NA	NA
scaffold727_G003	NA	NA
scaffold727_G004	NA	NA
scaffold758_G001_TNMD	TNMD	tenomodulin
scaffold784_G001_CXorf58	CXorf58	chromosome X open reading frame 58
scaffold806_G001	NA	NA
scaffold809_G001_KIAA2022	KIAA2022	KIAA2022
scaffold817_G001	NA	NA
scaffold829_G001_ELK1	ELK1	ELK1, ETS transcription factor
scaffold829_G002_CFP	CFP	complement factor properdin
scaffold832_G001_VSIG1	VSIG1	V-set and immunoglobulin domain containing 1
scaffold860_G001	NA	NA
scaffold861_G001_OR5M9	OR5M9	olfactory receptor family 5 subfamily M member 9
scaffold880_G001_NDUFB11	NDUFB11	NADH:ubiquinone oxidoreductase subunit B11
scaffold880_G002_RBM10	RBM10	RNA binding motif protein 10
scaffold913_G001_MAOB	MAOB	monoamine oxidase B
scaffold927_G001_PPP1R2P9	PPP1R2P9	protein phosphatase 1 regulatory inhibitor subunit 2 pseudogene 9
scaffold966_G001_TRA2A	TRA2A	transformer-2 protein homolog alpha
scaffold975_G001_NDP	NDP	NDP, norrin cystine knot growth factor
scaffold978_G001_TBC1D26	TBC1D26	TBC1 domain family member 26
scaffold988_G001_SPRY3	SPRY3	sprouty RTK signaling antagonist 3
scaffold992_G001	NA	NA
scaffold993_G001	NA	NA

Table S9. The distribution of InDels in the Marco Polo Sheep genome.

Location	Count
Intergenetic	231,841
Intron	125,007
Exonic	
Frameshift	465
Inframe	163
**Total**	**384,018**

Table S10. Prediction of repetitive elements in the assembled Marco Polo Sheep genome.

Type	Repeat Size (bp)	% of genome
**TRF**	23,720,137	0.87
**RepeatMasker**	1,151,260,621	42.46
**RepeatProteinMask**	485,793,737	17.92
De novo	1,006,131,246	37.11
**Total**	1,266,772,619	46.72

Table S11. Classification of interspersed repeats in the assembled Marco Polo Sheep genome.

Type[1]	Length (bp)	% of repeat	% of genome
**SINE**	304,404,425	24.09	11.23
**LINE**	915,778,362	72.48	33.78
L1	343,943,304	27.22	12.69
L2	60,852,671	4.82	2.24
**LTR**	145,757,824	11.54	5.38
Copia	48,057	0.00	0.00
Gypsy	2,145,882	0.17	0.08
**DNA**	67,331,380	5.33	2.48
hAT-Charlie	31,841,761	2.52	1.17
TcMar-Tigger	15,516,387	1.23	0.57
hAT-Tip100	7,570,498	0.60	0.28
**Unclassified**	3,353,047	0.27	0.12
**Satellites**	40,447,045	3.20	1.49
**Simple repeats**	37,774,449	2.99	1.39
**Small RNA**	103,014	0.01	0.00
**Total repeats**	**1,263,514,895**	**100**	**46.60**
**Low complexity sequence**	3,970,379	-	0.15

[1]All repeat types were assigned according to homology to the Repbase database (http://www.girinst.org/repbase).

Table S12. Data on all species used during the genome analysis.

Species	Number of gene	Version	Number of original genes
**Cattle (*Bos Taurus*)** [1]	19,948	UMD3.1	19,994
**Dog, (*Canis familiaris*)** [1]	19,770	CanFam3.1	19,856
**Goat (*Capra hircus*)** [1]	22,074	CHIR_1.0	22,172
**Human (*Homo sapiens*)** [1]	22,286	GRCh38	23,043
**Horse (*Equus caballu*)** [1]	20,359	EquCab2	20,449
**Marco Polo Sheep (*Ovis ammon polii*)**	20,336	-	20,336
**Opossum (*Monodelphis domestica*)** [1]	21,272	BROADO5	21,327
**Pig (*Sus scrofa*)** [1]	21,512	Sscrofa10.2	21,630
**Sheep (*Ovis aries*)** [2]	20,864	Oar_v3.1	20,921

[1]Genome annotations were download from ensembl release 88

[2]Goat genome annotations was download from http://gigadb.org/dataset/100082

Table S13. Prediction of protein-coding genes in the Marco Polo Sheep.

Gene set		Total Genes Predicted	Average Gene Length (bp)	Average CDS Length (bp)	Average Exons per Gene	Average Exon Length (bp)	Average Intron Length (bp)
	**Augustus**	23,602	44,982.71	1,434.66	8.17	175.60	6,053.96
De novo	**geneid**	52,948	37,557.96	1,051.23	7.59	138.49	5,539.15
	**GenScan**	22,446	37,784.50	1,351.55	8.08	167.20	5,143.46
	Bos taurus	23,901	22,959.65	1,252.31	6.60	189.83	4,017.29
**Homolog**	***Equus caballus***	22,646	22,955.46	1,223.79	6.51	187.95	4,090.15
	Homo sapiens	22,730	27,969.09	1,317.80	6.85	192.38	4,743.39
	Ovis aries	26,305	21,311.19	1,170.84	6.24	187.77	4,014.51
	Sus scrofa	24,207	18,286.98	1,121.31	5.81	192.92	3,675.95
**EVM**	--	22,474	38,494.41	1,436.76	8.24	174.46	5,121.64
**Final set**	--	20,336	43,765.40	1,671.37	9.67	172.89	4,856.65

Table S14. Comparative gene statistics.

Gene set	Numbers	Average Gene Length (bp)	Average CDS Length (bp)	Average Exons per Gene	Average Exon Length (bp)	Average Intron Length (bp)
Ovis ammon polii	20,336	43,765.40	1,671.37	9.67	172.89	4,856.65
Ovis aries	20,921	38,827.55	1,552.55	9.59	161.82	3,984.87
Capra hircus	22,172	29,969.00	1,384.96	8.23	168.36	3,955.55
Bos taurus	19,994	39,064.53	1,597.24	9.56	167.07	3,950.71
Homo sapiens	19,636	63,309.52	1,646.42	9.62	171.23	5,796.88

The genome versions: *Ovis aries* (Oar_v3.1), *Capra hircus* (CHIR_1.0), *Bos Taurus* (UMD3.1) and *Homo sapiens* (GRCh38).

Table S15. Functional annotation of predicted genes in the Marco Polo Sheep.

	Database	Number	Percent (%)
**Total**		20,336	100
	**InterPro**	17,726	87.17
	**GO**	14,436	70.99
**Annotated**	**Swiss-Prot**	18,642	91.67
	**TrEMBL**	18,777	92.33
	**KEGG**	11,643	57.25
**Unannotated**		1,515	7.45

Table S16. Summary statistics of non-coding RNAs in the Marco Polo Sheep.

Type		Copy number	Average length(bp)	Total length(bp)	% of genome
miRNA		684	84.88	58,056	0.00%
tRNA		836	73.46	61,410	0.00%
rRNA		277	124.51	34,488	0.00%
	5S	267	113.79	30,383	0.00%
	5.8S	6	148.50	891	0.00%
snRNA		1,181	115.65	136,578	0.01%
	CD-box	121	104.71	12,670	0.00%
	HACA-box	232	136.39	31,642	0.00%
	splicing	828	111.43	92,266	0.00%

Table S17. Summary of synteny alignments.

	Marco Polo Sheep - Sheep[1]	Marco Polo Sheep - Goat[1]	Sheep - Goat[1]
Query aligned length[2]	2,289,207,691 (91.37%)	2,296,815,924 (91.68%)	2,396,049,695 (92.69%)
Reference aligned length[2]	2,288,785,514 (88.55%)	2,296,974,846 (88.96%)	2,396,537,135 (92.81%)
Autosome aligned sites[3]	2,196,120,340 (89.65%)	2,216,550,854 (89.88%)	2,286,240,691 (92.70%)
X-chromosome aligned sites[3]	89,339,586 (66.09%)	73,079,892 (63.03%)	101,232,147 (87.31%)
Total aligned sites[3]	2,285,459,926	2,289,630,746	2,387,472,838
Total different sites	16,215,677	48,769,546	52,055,499
Percent divergence	0.00738	0.02200	0.02277

Goat genome version is ARS1 and sheep genome version is Oar_v3.1.

[1]The combination with the following format "Query-Refernece".

[2]Calculated the aligned length including the small InDels. The percent represent the proportion of the aligned length/corresponded type length.

[3]Calculated the aligned sites without the small InDels. The percent represent the proportion of the aligned length/reference type length.

Table S18. Summary of breakpoints between Marco Polo Sheep, sheep and goat.

Type of breakpoints	Marco Polo Sheep - Sheep	Marco Polo Sheep - Goat	Sheep - Goat
Inter-chromosomal	8,303	3,080	6,431
Intra-chromosomal	2,871	2,184	4,033
Inversion	582	762	1,222
**Total**	**11,756**	**6,026**	**11,686**

Goat genome version is ARS1 and sheep genome version is Oar_v3.1.

Table S19. Summary statistics of gene families in 9 species.

Species	Total genes	Genes in families	Unclustered genes	Families	Unique families	Genes per family	Maximum gene family size
**Cattle**	19,948	19,192	756	15,018	19	1.28	68
**Dog**	19,770	18,161	1,609	14,445	37	1.26	46
**Goat**	22,074	18,718	3,356	15,184	17	1.23	36
**Human**	22,286	21,603	683	14,864	136	1.45	339
**Horse**	20,359	19,658	701	14,383	59	1.37	514
**Marco Polo Sheep**	20,336	17,247	3,089	14,062	50	1.23	45
**Opossum**	21,272	19,235	2,037	13,379	132	1.44	484
**Pig**	21,512	18,733	2,779	13,733	59	1.36	50
**Sheep**	20,864	19,415	1,449	15,354	38	1.26	47
**All**	188,421	171,962	16,459	17,576	-	9.78	767

Genome versions: Cattle (UMD3.1), Dog (CanFam3.1), Goat (CHIR_1.0), Human (GRCh38), Horse (EquCab2), Opossum (BROADO5), Pig (Sscrofa10.2), Sheep (Oar_v3.1).

Table S20. GO enrichment analysis of the expanded gene families in the Marco Polo Sheep lineage.

GO	Type	Function	Adjust P value
GO:0007156	biological process	homophilic cell adhesion via plasma membrane adhesion molecules	1.57E-24
GO:0016337	biological process	single organismal cell-cell adhesion	2.21E-21
GO:0007186	biological process	G-protein coupled receptor signaling pathway	1.47E-15
GO:0007155	biological process	cell adhesion	5.82E-13
GO:0022610	biological process	biological adhesion	5.82E-13
GO:0007166	biological process	cell surface receptor signaling pathway	1.35E-11
GO:0009987	biological process	cellular process	5.92E-07
GO:0055085	biological process	transmembrane transport	1.05E-05
GO:0050896	biological process	response to stimulus	0.001710055
GO:0007165	biological process	signal transduction	0.004292866
GO:0006457	biological process	protein folding	0.004883729
GO:0008150	biological process	biological_process	0.021982392
GO:0005886	cellular component	plasma membrane	1.29E-19
GO:0005575	cellular component	cellular_component	1.89E-16
GO:0044425	cellular component	membrane part	1.38E-14
GO:0016021	cellular component	integral component of membrane	2.23E-14
GO:0031224	cellular component	intrinsic component of membrane	3.53E-14
GO:0004984	molecular function	olfactory receptor activity	1.06E-30
GO:0004930	molecular function	G-protein coupled receptor activity	2.48E-19
GO:0038023	molecular function	signaling receptor activity	2.28E-15
GO:0004888	molecular function	transmembrane signaling receptor activity	2.14E-14
GO:0004871	molecular function	signal transducer activity	1.54E-13
GO:0060089	molecular function	molecular transducer activity	1.54E-13
GO:0004872	molecular function	receptor activity	2.01E-13
GO:0042626	molecular function	ATPase activity, coupled to transmembrane movement of substances	1.19E-08
GO:0015399	molecular function	primary active transmembrane transporter activity	3.47E-08
GO:0015405	molecular function	P-P-bond-hydrolysis-driven transmembrane transporter activity	3.47E-08
GO:0016820	molecular function	hydrolase activity, acting on acid anhydrides, catalyzing transmembrane movement of substances	4.27E-08
GO:0043492	molecular function	ATPase activity, coupled to movement of substances	4.31E-07
GO:0022804	molecular function	active transmembrane transporter activity	1.77E-06
GO:0042623	molecular function	ATPase activity, coupled	0.001810539
GO:0046982	molecular function	protein heterodimerization activity	0.00432788
GO:0051082	molecular function	unfolded protein binding	0.004364718
GO:0005509	molecular function	calcium ion binding	0.008420616
GO:0008131	molecular function	primary amine oxidase activity	0.013909209
GO:0005215	molecular function	transporter activity	0.01437283

GO:0016620	molecular function	oxidoreductase activity, acting on the aldehyde or oxo group of donors, NAD or NADP as acceptor	0.022230804
GO:0004457	molecular function	lactate dehydrogenase activity	0.028854215
GO:0004459	molecular function	L-lactate dehydrogenase activity	0.028854215

Table S21. Candidate PSGs in the Marco Polo Sheep lineage.

Marco Polo Sheep gene id	Gene symbol	Gene name	P value
scaffold36_G095_SCN3B	*SCN3B*	sodium voltage-gated channel beta subunit 3	0
scaffold107_G075_COL16A1	*COL16A1*	collagen type XVI alpha 1 chain	0
scaffold107_G069_FABP3	*FABP3*	fatty acid binding protein 3	0
scaffold61_G011_CYP11A1	*CYP11A1*	cytochrome P450 family 11 subfamily A member 1	0
scaffold61_G010_CCDC33	*CCDC33*	coiled-coil domain containing 33	0
scaffold18_G191_KMT2B	*KMT2B*	lysine methyltransferase 2B	0
scaffold10_G184	NA	NA	0
scaffold71_G019_ZBTB17	*ZBTB17*	zinc finger and BTB domain containing 17	0
scaffold147_G022_SRSF7	*SRSF7*	serine and arginine rich splicing factor 7	0
scaffold67_G074_ELSPBP1	*ELSPBP1*	epididymal sperm binding protein 1	0
scaffold15_G309_YBX2	*YBX2*	Y-box binding protein 2	0
scaffold135_G009_TPM2	*TPM2*	tropomyosin 2 (beta)	0
scaffold47_G066_PNPLA1	*PNPLA1*	patatin like phospholipase domain containing 1	0
scaffold76_G030_ATP10B	*ATP10B*	ATPase phospholipid transporting 10B	0
scaffold91_G038_ATF7	*ATF7*	activating transcription factor 7	0
scaffold27_G090_PLET1	*PLET1*	placenta expressed transcript 1	0
scaffold380_G019_SMPD4	*SMPD4*	sphingomyelin phosphodiesterase 4	0
scaffold78_G055_OBSL1	*OBSL1*	obscurin like 1	0
scaffold5_G014_MCF2L2	*MCF2L2*	MCF.2 cell line derived transforming sequence-like 2	0
scaffold18_G207_WDR62	*WDR62*	WD repeat domain 62	1.00E-09
scaffold48_G065_LRGUK	*LRGUK*	leucine rich repeats and guanylate kinase domain containing	1.00E-09
scaffold445_G016_P2RX3	*P2RX3*	purinergic receptor P2X 3	1.00E-09
scaffold167_G008_SAMSN1	*SAMSN1*	SAM domain, SH3 domain and nuclear localization signals 1	1.00E-09
scaffold155_G004_ROBO1	*ROBO1*	roundabout guidance receptor 1	1.00E-09
scaffold10_G299_MCM2	*MCM2*	minichromosome maintenance complex component 2	2.00E-09
scaffold436_G005_MTM1	*MTM1*	myotubularin 1	2.00E-09
scaffold149_G035_CCL22	*CCL22*	C-C motif chemokine ligand 22	3.00E-09
scaffold263_G011_SYT1	*SYT1*	synaptotagmin 1	3.00E-09
scaffold26_G086_C2CD2	*C2CD2*	C2 calcium dependent domain containing 2	4.00E-09
scaffold72_G036_PDLIM3	*PDLIM3*	PDZ and LIM domain 3	1.10E-08
scaffold52_G017_IKBKB	*IKBKB*	inhibitor of nuclear factor kappa B kinase subunit beta	1.20E-08
scaffold174_G040_CIRBP	*CIRBP*	cold inducible RNA binding protein	1.20E-08
scaffold296_G005_HYAL4	*HYAL4*	hyaluronoglucosaminidase 4	1.40E-08
scaffold168_G033_IKZF5	*IKZF5*	IKAROS family zinc finger 5	1.40E-08
scaffold37_G021_C8orf34	*C8orf34*	chromosome 8 open reading frame 34	1.50E-08
scaffold276_G029_FCER2	*FCER2*	Fc fragment of IgE receptor II	1.90E-08
scaffold161_G073_SMG6	*SMG6*	SMG6, nonsense mediated mRNA decay factor	4.20E-08
scaffold219_G006_GATAD1	*GATAD1*	GATA zinc finger domain containing 1	6.60E-08
scaffold33_G048_CRY1	*CRY1*	cryptochrome circadian clock 1	7.60E-08
scaffold537_G003_RIBC1	*RIBC1*	RIB43A domain with coiled-coils 1	1.10E-07

scaffold249_G064_LRP1	*LRP1*	LDL receptor related protein 1	1.76E-07
scaffold108_G065_LMCD1	*LMCD1*	LIM and cysteine rich domains 1	2.04E-07
scaffold244_G010_TAF2	*TAF2*	TATA-box binding protein associated factor 2	2.64E-07
scaffold68_G121_PES1	*PES1*	pescadillo ribosomal biogenesis factor 1	3.24E-07
scaffold18_G242_RYR1	*RYR1*	ryanodine receptor 1	3.32E-07
scaffold2_G236_REXO5	*REXO5*	RNA exonuclease 5	4.40E-07
scaffold380_G029_SNAP29	*SNAP29*	synaptosome associated protein 29	4.42E-07
scaffold69_G080_NOXO1	*NOXO1*	NADPH oxidase organizer 1	6.16E-07
scaffold39_G066_PXDC1	*PXDC1*	PX domain containing 1	6.86E-07
scaffold4_G263_C5orf15	*C5orf15*	chromosome 5 open reading frame 15	7.17E-07
scaffold184_G064_CELA1	*CELA1*	chymotrypsin like elastase family member 1	7.41E-07
scaffold6_G035_RNASEL	*RNASEL*	ribonuclease L	9.25E-07
scaffold23_G030	NA	NA	1.02E-06
scaffold39_G067_SLC22A23	*SLC22A23*	solute carrier family 22 member 23	1.09E-06
scaffold42_G098_GPSM2	*GPSM2*	G-protein signaling modulator 2	1.10E-06
scaffold69_G079_TBL3	*TBL3*	transducin beta like 3	1.30E-06
scaffold281_G001_DPH6	*DPH6*	diphthamine biosynthesis 6	1.30E-06
scaffold174_G026_ARID3A	*ARID3A*	AT-rich interaction domain 3A	1.38E-06
scaffold14_G064_SETDB2	*SETDB2*	SET domain bifurcated 2	1.47E-06
scaffold138_G026_TOX	*TOX*	thymocyte selection associated high mobility group box	1.57E-06
scaffold80_G033_C12orf71	*C12orf71*	chromosome 12 open reading frame 71	1.67E-06
scaffold327_G043_GPAA1	*GPAA1*	glycosylphosphatidylinositol anchor attachment 1	1.98E-06
scaffold29_G146_KDM5A	*KDM5A*	lysine demethylase 5A	2.36E-06
scaffold37_G002_MTFR1	*MTFR1*	mitochondrial fission regulator 1	3.05E-06
scaffold6_G080_PROX1	*PROX1*	prospero homeobox 1	3.78E-06
scaffold81_G038_HSD3B1	*HSD3B1*	3 beta-hydroxysteroid dehydrogenase/Delta 5-->4-isomerase type 1	4.29E-06
scaffold478_G009_ARHGAP30	*ARHGAP30*	Rho GTPase activating protein 30	5.26E-06
scaffold18_G127_ANKRD27	*ANKRD27*	ankyrin repeat domain 27	5.52E-06
scaffold104_G038_VPS50	*VPS50*	VPS50, EARP/GARPII complex subunit	6.61E-06
scaffold28_G016_AFDN	*AFDN*	afadin, adherens junction formation factor	6.69E-06
scaffold22_G195_WDR45B	*WDR45B*	WD repeat domain 45B	7.31E-06
scaffold126_G006_CDAN1	*CDAN1*	codanin 1	8.02E-06
scaffold298_G002_CTTNBP2	*CTTNBP2*	cortactin binding protein 2	8.05E-06
scaffold518_G010_SH3BP5L	*SH3BP5L*	SH3 binding domain protein 5 like	8.65E-06
scaffold220_G025_TTC21A	*TTC21A*	tetratricopeptide repeat domain 21A	8.69E-06
scaffold255_G007_NUDT6	*NUDT6*	nudix hydrolase 6	9.73E-06
scaffold25_G073_OTUD7B	*OTUD7B*	OTU deubiquitinase 7B	1.14E-05
scaffold199_G005_GIN1	*GIN1*	gypsy retrotransposon integrase 1	2.77E-05
scaffold53_G117_STX16	*STX16*	syntaxin 16	3.04E-05
scaffold784_G005_PRDX4	*PRDX4*	peroxiredoxin 4	3.17E-05
scaffold55_G060_FASTKD1	*FASTKD1*	FAST kinase domains 1	3.57E-05
scaffold80_G043_FAR2	*FAR2*	fatty acyl-CoA reductase 2	4.30E-05
scaffold129_G006_CHST9	*CHST9*	carbohydrate sulfotransferase 9	4.60E-05

scaffold25_G065_RPRD2	*RPRD2*	regulation of nuclear pre-mRNA domain containing 2	5.43E-05
scaffold162_G028_GNRH1	*GNRH1*	gonadotropin releasing hormone 1	5.63E-05
scaffold78_G001_PECR	*PECR*	peroxisomal trans-2-enoyl-CoA reductase	7.04E-05
scaffold92_G016_SH3GL2	*SH3GL2*	SH3 domain containing GRB2 like 2, endophilin A1	7.58E-05
scaffold198_G002_RGS18	*RGS18*	regulator of G-protein signaling 18	0.0001143
scaffold3_G017_INHBA	*INHBA*	inhibin beta A subunit	0.0001291
scaffold33_G018_IGF1	*IGF1*	insulin like growth factor 1	0.0001734
scaffold26_G014_RIPPLY3	*RIPPLY3*	ripply transcriptional repressor 3	0.0001926
scaffold110_G006_LARP7	*LARP7*	La ribonucleoprotein domain family member 7	0.0001966
scaffold40_G077_MAT2A	*MAT2A*	methionine adenosyltransferase 2A	0.0002008
scaffold1350_G001_PHF6	*PHF6*	PHD finger protein 6	0.0002798
scaffold104_G014_SDHAF3	*SDHAF3*	succinate dehydrogenase complex assembly factor 3	0.0003251
scaffold71_G034_C1QTNF12	*C1QTNF12*	C1q and tumor necrosis factor related protein 12	0.0003295
scaffold43_G020_LRRC9	*LRRC9*	Leucine-rich repeat-containing protein 9	0.0004389
scaffold21_G022_PRRC1	*PRRC1*	proline rich coiled-coil 1	0.0004636
scaffold219_G008_ANKIB1	*ANKIB1*	ankyrin repeat and IBR domain containing 1	0.0004657
scaffold29_G013_ARHGAP8	*ARHGAP8*	Rho GTPase activating protein 8	0.0004864
scaffold6_G011_ABL2	*ABL2*	ABL proto-oncogene 2, non-receptor tyrosine kinase	0.0005076
scaffold324_G050_ZNF45	*ZNF45*	zinc finger protein 45	0.000532
scaffold218_G042_CPT1B	*CPT1B*	carnitine O-palmitoyltransferase 1, muscle isoform	0.0005381
scaffold31_G019_SPTLC3	*SPTLC3*	serine palmitoyltransferase long chain base subunit 3	0.0005727
scaffold236_G011_MEP1B	*MEP1B*	meprin A subunit beta	0.0006266
scaffold15_G346_SCIMP	*SCIMP*	SLP adaptor and CSK interacting membrane protein	0.0006518
scaffold258_G007_PDS5B	*PDS5B*	PDS5 cohesin associated factor B	0.000724
scaffold68_G045_MMAB	*MMAB*	methylmalonic aciduria (cobalamin deficiency) cblB type	0.0007252
scaffold35_G088_NPAS2	*NPAS2*	neuronal PAS domain protein 2	0.0007504
scaffold44_G024_C9orf135	*C9orf135*	chromosome 9 open reading frame 135	0.0007838
scaffold225_G006_AGL	*AGL*	amylo-alpha-1, 6-glucosidase, 4-alpha-glucanotransferase	0.0007882
scaffold128_G037_OGFRL1	*OGFRL1*	opioid growth factor receptor like 1	0.0007992
scaffold125_G005_ARMC3	*ARMC3*	armadillo repeat containing 3	0.0008529
scaffold4_G026_PRC1	*PRC1*	protein regulator of cytokinesis 1	0.0009371
scaffold163_G001_RIDA	*RIDA*	reactive intermediate imine deaminase A homolog	0.0009568
scaffold105_G027_MMS19	*MMS19*	MMS19 homolog, cytosolic iron-sulfur assembly component	0.0010023
scaffold424_G011_RGS12	*RGS12*	regulator of G-protein signaling 12	0.0010069
scaffold35_G063_M1AP	*M1AP*	meiosis 1 associated protein	0.001078
scaffold17_G007_PNPLA8	*PNPLA8*	patatin like phospholipase domain containing 8	0.0011398
scaffold26_G062_DNMT3L	*DNMT3L*	DNA methyltransferase 3 like	0.001254
scaffold17_G100_LIAS	*LIAS*	lipoic acid synthetase	0.001381
scaffold62_G077_HP1BP3	*HP1BP3*	heterochromatin protein 1 binding protein 3	0.0013891
scaffold75_G046_GPNMB	*GPNMB*	glycoprotein nmb	0.0014299
scaffold174_G016_PALM	*PALM*	paralemmin	0.0015357

scaffold24_G034_HNMT	*HNMT*	histamine N-methyltransferase	0.0015446
scaffold217_G001_DEPDC1B	*DEPDC1B*	DEP domain containing 1B	0.0015703
scaffold107_G036_DNAJC8	*DNAJC8*	DnaJ heat shock protein family (Hsp40) member C8	0.0017959
scaffold21655_G001_DUT	*DUT*	deoxyuridine triphosphatase	0.0018277
scaffold107_G074_PEF1	*PEF1*	penta-EF-hand domain containing 1	0.0018711
scaffold122_G003_HAS2	*HAS2*	hyaluronan synthase 2	0.0019278
scaffold21_G156_DENND1C	*DENND1C*	DENN domain containing 1C	0.0019867
scaffold169_G007_SERBP1	*SERBP1*	SERPINE1 mRNA binding protein 1	0.0020643
scaffold7_G094_EIF4E	*EIF4E*	eukaryotic translation initiation factor 4E	0.0021353
scaffold219_G024_C7orf57	*C7orf57*	chromosome 7 open reading frame 57	0.0028986
scaffold81_G009_DLGAP1	*DLGAP1*	DLG associated protein 1	0.0029248
scaffold139_G006_GHITM	*GHITM*	growth hormone inducible transmembrane protein	0.0030062
scaffold69_G166_ANKS3	*ANKS3*	ankyrin repeat and sterile alpha motif domain containing 3	0.0030146
scaffold184_G010_KANSL2	*KANSL2*	KAT8 regulatory NSL complex subunit 2	0.0031521
scaffold187_G040_ARHGEF17	*ARHGEF17*	Rho guanine nucleotide exchange factor 17	0.0033461
scaffold39_G037_MAK	*MAK*	male germ cell associated kinase	0.0036218
scaffold120_G017_CLRN1	*CLRN1*	clarin 1	0.0036672
scaffold36_G010_PPFIBP2	*PPFIBP2*	PPFIA binding protein 2	0.0039778
scaffold367_G010_SHC3	*SHC3*	SHC adaptor protein 3	0.0040239
scaffold102_G037_SH3PXD2B	*SH3PXD2B*	SH3 and PX domains 2B	0.0040993
scaffold152_G011_E2F4	*E2F4*	E2F transcription factor 4	0.0043781
scaffold13_G130_TMEM38B	*TMEM38B*	transmembrane protein 38B	0.0045179
scaffold149_G022_CNGB1	*CNGB1*	cyclic nucleotide gated channel beta 1	0.0045248
scaffold136_G032_IDE	*IDE*	insulin degrading enzyme	0.0046444
scaffold185_G023_ASTN1	*ASTN1*	astrotactin 1	0.0053684
scaffold113_G016_SMARCD3	*SMARCD3*	SWI/SNF related, matrix associated, actin dependent regulator of chromatin, subfamily d, member 3	0.0058774
scaffold104_G007_MIOS	*MIOS*	meiosis regulator for oocyte development	0.006044
scaffold21_G115_PLIN4	*PLIN4*	perilipin 4	0.0066212
scaffold240_G002_PERP	*PERP*	PERP, TP53 apoptosis effector	0.0069755
scaffold62_G125_NECAP2	*NECAP2*	NECAP endocytosis associated 2	0.0070838
scaffold271_G009_FAP	*FAP*	fibroblast activation protein alpha	0.0071117
scaffold26_G065_TRAPPC10	*TRAPPC10*	trafficking protein particle complex 10	0.0074111
scaffold15_G245_MYH13	*MYH13*	myosin heavy chain 13	0.0074222
scaffold129_G027_ANKRD29	*ANKRD29*	ankyrin repeat domain 29	0.0080622
scaffold47_G005_GCLC	*GCLC*	glutamate-cysteine ligase catalytic subunit	0.0113879
scaffold26_G056_LRRC3	*LRRC3*	leucine rich repeat containing 3	0.011798
scaffold283_G007_SPATA4	*SPATA4*	spermatogenesis associated 4	0.0129075
scaffold4_G024_UNC45A	*UNC45A*	unc-45 myosin chaperone A	0.0131852
scaffold29_G030_SERHL2	*SERHL2*	serine hydrolase-like 2	0.0182583
scaffold17_G113_PGM2	*PGM2*	phosphoglucomutase 2	0.0197053
scaffold71_G083_MEGF6	*MEGF6*	multiple EGF like domains 6	0.0211719
scaffold119_G002_BCDIN3D	*BCDIN3D*	BCDIN3 domain containing RNA methyltransferase	0.024793

scaffold40_G038_CKAP2L	*CKAP2L*	cytoskeleton associated protein 2 like	0.0285168
scaffold36_G069_RRAS2	*RRAS2*	related RAS viral (r-ras) oncogene homolog 2	0.0329742

154
