# Supplementary material for: Draft genome of the Marco Polo Sheep (Ovis ammon polii)
Source: Gigascience. 2017 Nov 1;6(12):1–7. doi: 10.1093/gigascience/gix106 (PMC5740985; doi:10.1093/gigascience/gix106)
Supplement: Response-to-Reviewer-Comments_Original-Submission.pdf [file gix106_response-to-reviewer-comments_original-submission.pdf]

Author's Response To Reviewer Comments

Response to Referees
Your manuscript "The genome of the Marco Polo Sheep (Ovis ammon polii)" (GIGA-D-17-00160) has been assessed by our reviewers. Based on these reports, and my own assessment as Editor, I am pleased to inform you that it is potentially acceptable for publication in GigaScience, once you have carried out some essential revisions suggested by our reviewers. Their reports are below.
The main points of the reviewers concern places in the manuscript where your wording should be more exact, or where you should be more careful with your interpretations (for example, the statement on effective population size, based on variation in just a single animal, may not be warranted, as pointed out by reviewer 1).
Reply: We are grateful for the editor and reviewers' helpful suggestions and instructions.
I note that you indicate several corresponding authors. OUP has a policy of only taking one - most responsive - author as corresponding author. The definition of corresponding authorship is one of responsiveness rather than seniority. The corresponding author is the one individual who takes primary responsibility for communication with the journal during the manuscript submission, peer review, and publication process. Please refer to the information below on our homepage and please decide based on these guidelines who should be corresponding author.
Reply: Nowadays most genome projects are results of collaborative results of several groups. In this work, all the three corresponding authors were made significant contributions in designing the project and organizing the manuscript, and thus we listed three correspondent authors with Dr. Kun Wang as the first correspondent author to communicate with the editorial office. We have listed the detail contributions in the section of Authors' contributions in the revised manuscript, which is highlighted with yellow.
You indicate several "co-first" authors with equal contributions. Please explain in more detail in how far the contributions of all three first authors are exactly equal. See also the CASRAI guidelines to define authorship roles:
http://dictionary.casrai.org/Contributor_Roles
Reply: All of three "co-first" authors were the major material collectors and data analysts, and have taken part in the paper's writing in this work. We have classified their works and listed the detail contribution of all authors in the section of Authors' contributions in the revised manuscript. Those three authors were evaluated and each of them should be listed as "co-first" authors.

Reviewer #1: The manuscript describes the construction of a draft assembly of the Marco Polo Sheep to an equivalent standard to the sheep, goat and cattle genomes. The assembly parameters are in line with what can reasonably be expected for the approach used. The text adequately documents the assembly and analysis methodologies. The analysis results are also consistent with expectations.
However, I think that the use of variation in a single animal to make such a strong statement about effective population size, and endangered population, of Marco Polo Sheep may not be warranted (lines 145-146).
Reply: We appreciate the reviewer's positive comments on this work. We agree that only one single sample could not represent the population size of a species. We have therefore removed the related statement, and furthermore we have added PSMC analysis to provide estimation on

the demographic history, which is a highly reliable method to estimate demographic history using a single genome data.

I am also concerned about Table S21, where enrichment of potentially selected genes in GO category is shown. The p-values shown are marginal for p-values adjusted for multiple testing (I generally disregard adjusted p-values of >10-3). However unlike other tables the p-values are not indicated as adjusted. If they are not adjusted the table should be omitted and the wording altered at lines 252/3. I note that "enriched" rather than "significantly enriched" is used in the text, but readers will tend to assume "significantly enriched" from such a mention in the text even if it is not stated that way.

Reply: Thanks for the suggestion, and we agree that the enrichment significance was marginal and thus we have replaced the GO category enrichment result with a paragraph to discuss some specific PSGs to make sure that all conclusions in the manuscript are solid.

Line 57, "long horns" rather than "long horn"

Reply: Corrected as suggested.

Lines 110/111, it is not clear exactly which versions of the sheep and goat genomes are being used here, one would have to check the references to find out, whereas elsewhere specific versions are mentioned in the text. I suggest that assembly version designations consistent with those used elsewhere are included here.

Reply: We apologize for causing this confusion and have added the information of the published genomes' versions that we used in our analysis in the revised manuscript.

Line 130 two goat assemblies are mentioned without references. How do they relate to each other and which assembly is being used on other occasions where only one goat genome assembly is mentioned? This is not clear in a number of the tables and at places in the text.

Reply: We have added the information of corresponding references, relationship and which version we have used in this revised manuscript.

Reviewer #2: This manuscript describes the genome sequencing and assembly of Marco Polo sheep (Ovis ammon poliii) a wild sheep species that is interesting in several regards. First, the species is adapted to living at high altitude. Second, the species is of conservation concern due to hunting, habitat loss and a limited range. Finally, there remains some uncertainty as to how often and where sheep were domesticated, and O. ammon species may have contributed to the genomes of modern domestic sheep.

In many ways, the manuscript is fairly routine, in the sense that the authors describe a shotgun sequencing experiment, subsequent assembly of the data and some comparative analyses of Marco Polo sheep and domesticated ovids. The work appears to have been conducted to a high standard (I lack the expertise to really judge the assembly and annotation methodologies), and it certainly looks to be thorough. It is notable that considerably more sequence data were collected (>1000 Gb) than for the assembly of the domestic sheep genome, where ~220 Gbp and ~155 Gbp of two Texel sheep was generated (Jiang et al. 2014, Science 344:1168). The N50 contig length is a little lower than that of the domestic sheep genome (~30Kb and ~40Kb respectively). Overall, the manuscript contains an impressive amount of data, it is well written, and the resource will be of interest to animal geneticists.

Reply: We are grateful for these positive comments. The N50 contig length of NGS genome assembly could be influenced by many factors, including the strategies of library construction, gap-filling methods and the coverage of Pair-end (PE) libraries (usually with an insert length less than 2kb). There is not much difference between the amount of PE reads used in the assembly

and gap-filling of Macro Polo sheep (425.29 Gb clean data) and domestic sheep (374.8 Gb of Illumina sequencing data but with 9 Gb of Roche 454 long sequencing reads data). The N50 contig length of the Marco Polo sheep is slightly shorter than that of published domestic sheep perhaps because the domestic sheep genome project used long reads Roche 454 data, which is now not available in the market. Besides the N50 contig length of 22 NGS mammalian assemblies ranges from 16 to 66 Kb (Table S3 of Wang et al. 2017, GigaScience 6 (4), 1-5), indicating the N50 contig length we obtained here is fairly within the expectation for a mammalian species.

Unsurprisingly, most of the comparisons between Marco Polo sheep and domestic sheep show very high conservation of synteny, GC content, gene number, etc. Given that these analyses are conducted independently of those done in domestic sheep, the similarity can be seen as reassurance that the analyses conducted on Marco Polo sheep genome are robust and reliable. One slight discrepancy is the average intron length (Table S13); in Marco Polo sheep this appears to be ~20% larger than is seen in domestic sheep, goats and cattle. Of course, this could be a real feature of the Marco Polo sheep genome. The authors argue that intron length appears to be conserved across the mammals they study (lines 184-185), but it might be worth trying to establish why it appears to be longer in Marco Polo Sheep.

Reply: We thank the reviewer for raising this important issue. We checked the differences of intron length, exon length, etc. between Macro Polo sheep and domestic sheep, goat or human for each orthologous gene. Generally, these parameters were very close within these species (Fig. S7). The increasing of intron length in Macro Polo sheep was not very significant but did existed. More LINE sequences were found in the intron regions of Macro Polo sheep than sheep or goat, suggesting that transposon insertion might have contributed to intron length increase. We have added this discussion on page 10 highlighted with yellow.

Table S20 is useful because it presents a list of genes that are putatively under positive selection. It's a bit of a shame that these are not examined or even discussed in the context of adaptation to high altitude, especially as in the Conclusion the authors comment that the genome was partially sequenced because the species is a model for studying this very question. I understand that the primary aim of the paper is to describe the resource, but nonetheless, the results presented in Table S20 warrant some discussion.

Reply: Thank you for your suggestion, we have added a detailed paragraph on page 14 to describe the possible biological relevance of positively selected genes in high altitude adaptation, highlighted with yellow.

On lines 142-146, the relatively low nucleotide diversity of Marco Polo sheep relative to that seen in domestic sheep is discussed. However, the sampled male was bred and reared in a zoo, and very little is said about whether the diversity is likely to be similar to that seen in wild animals. For example, if the male was inbred, because there were relatively few unrelated captive animals, then the low diversity could be unrepresentative. If the authors know that the animal is not inbred, it could be worth them stating so. Inbreeding would be identifiable through e.g. Runs of Homozygosity.

Reply: We fully agree with the referee. The sampled male was originally captured from wild. However, the current available methods, such like Plink, bcftools, are unworkable for only one individual. Thus, we made a rough inference of RoH based on the distribution of heterozygosity along the genome with Hidden Markov model. We found the RoH regions (heterozygosity ratio $< 0.003\%$, 1/46 of average) were made up 14 % of the total 49,317 non-overlapping 50k window. It should be noted that this result should be interpreted with caution because this is only

from one individual. We have described more about this issue in the revised manuscript highlighted with yellow.

Minor typos:

1) Line 405 - 'Veen' diagram should be 'Venn'.

Reply: Sorry for the typo. Corrected as suggested.
