# Supplementary material for: Draft genome of the Marco Polo Sheep (Ovis ammon polii)
Source: Gigascience. 2017 Nov 1;6(12):1–7. doi: 10.1093/gigascience/gix106 (PMC5740985; doi:10.1093/gigascience/gix106)
Supplement: Response-to-Reviewer-Comments_Revision-1.pdf [file gix106_response-to-reviewer-comments_revision-1.pdf]

Author's Response To Reviewer Comments

In your next (final) version, please also remove any highlighting of changes that have been made for the purpose of peer review.
Reply: We have removed all highlighting in the manuscript.

Regarding the corresponding authors, we must insist that you list a maximum of two co-authors with this role, as explained in our instructions for authors.
Reply: We have reduced one corresponding author from the manuscript. Kun wang and Hongjiang wei were now listed as the corresponding authors.

I have only one minor comment, and that concerns the heterozygosity analysis. I think the authors are probably justified in including this section, because although it is based on just one animal, it is presumably possible for researchers studying other sheep breeds to perform similar analyses and make comparisons. I wonder whether the 14% of the genome that is highly homozygous - I think this is a better word to use than 'homogeneous' (line 152) - could be due to recent inbreeding, and that the remainder of the genome is more useful for comparing levels of diversity between Marco Polo sheep and other sheep? I'm not proposing that the authors analyse these regions in any more detail though.
Reply: Thank you for the suggestion, and we agree with that recent inbreeding could lead to the highly homozygous in the genome. We have re-written related sentences in line 143-148.
"The genomic regions with "low heterozygosity" state that made up 14% of the genome were highly homozygous (mean heterozygosity rate = 0.003%), which could be explained by either loss of polymorphism in endangered species [20] or recent inbreeding in some specific Macro Polo sheep individuals. More samples will be required to test whether the highly homozygous status was common in this species."
