# Supplementary material for: Draft genome of the Marco Polo Sheep (Ovis ammon polii)
Source: Gigascience. 2017 Nov 1;6(12):1–7. doi: 10.1093/gigascience/gix106 (PMC5740985; doi:10.1093/gigascience/gix106)
Supplement: Reviewer-1-Report-(Original-Submission).pdf [file gix106_reviewer-1-report-(original-submission).pdf]

Reviewer Report

Title: The genome of the Marco Polo Sheep (Ovis ammon polii)

Version: Original Submission **Date:** 7/13/2017

Reviewer name: Brian P. Dalrymple

Reviewer Comments to Author:

The manuscript describes the construction of a draft assembly of the Marco Polo Sheep to an equivalent standard to the sheep, goat and cattle genomes. The assembly parameters are in line with what can reasonably be expected for the approach used. The text adequately documents the assembly and analysis methodologies. The analysis results are also consistent with expectations.However, I think that the use of variation in a single animal to make such a strong statement about effective population size, and endangered population, of Marco Polo Sheep may not be warranted (lines 145-146).I am also concerned about Table S21, where enrichment of potentially selected genes in GO category is shown. The p-values shown are marginal for p-values adjusted for multiple testing (I generally disregard adjusted p-values of >10-3). However unlike other tables the p-values are not indicated as adjusted. If they are not adjusted the table should be omitted and the wording altered at lines 252/3. I note that "enriched" rather than "significantly enriched" is used in the text, but readers will tend to assume "significantly enriched" from such a mention in the text even if it is not stated that way.Line 57, "long horns" rather than "long horn"Lines 110/111, it is not clear exactly which versions of the sheep and goat genomes are being used here, one would have to check the references to find out, whereas elsewhere specific versions are mentioned in the text. I suggest that assembly version designations consistent with those used elsewhere are included here.Line 130 two goat assemblies are mentioned without references. How do they relate to each other and which assembly is being used on other occasions where only one goat genome assembly is mentioned? This is not clear in a number of the tables and at places in the text.

Level of Interest

Please indicate how interesting you found the manuscript: An article whose findings are important to those with closely related research interests

Quality of Written English

Please indicate the quality of language in the manuscript: Acceptable

Declaration of Competing Interests

Please complete a declaration of competing interests, considering the following questions:

- Have you in the past five years received reimbursements, fees, funding, or salary from an organisation that may in any way gain or lose financially from the publication of this manuscript, either now or in the future?
- Do you hold any stocks or shares in an organisation that may in any way gain or lose financially from the publication of this manuscript, either now or in the future?
- Do you hold or are you currently applying for any patents relating to the content of the manuscript?
- Have you received reimbursements, fees, funding, or salary from an organization that holds or has applied for patents relating to the content of the manuscript?
- Do you have any other financial competing interests?
- Do you have any non-financial competing interests in relation to this paper?

If you can answer no to all of the above, write 'I declare that I have no competing interests' below. If your reply is yes to any, please give details below.

I declare that I have no competing interests.

I agree to the open peer review policy of the journal. I understand that my name will be included on my report to the authors and, if the manuscript is accepted for publication, my named report including any attachments I upload will be posted on the website along with the authors' responses. I agree for my report to be made available under an Open Access Creative Commons CC-BY license (http://creativecommons.org/licenses/by/4.0/). I understand that any comments which I do not wish to be included in my named report can be included as confidential comments to the editors, which will not be published.

I agree to the open peer review policy of the journal
