# Supplementary material for: Draft genome of the Marco Polo Sheep (Ovis ammon polii)
Source: Gigascience. 2017 Nov 1;6(12):1–7. doi: 10.1093/gigascience/gix106 (PMC5740985; doi:10.1093/gigascience/gix106)
Supplement: Reviewer-2-Report-(Original-Submission).pdf [file gix106_reviewer-2-report-(original-submission).pdf]

Reviewer Report

Title: The genome of the Marco Polo Sheep (Ovis ammon polii)

Version: Original Submission **Date:** 7/17/2017

Reviewer name: Jon Slate

Reviewer Comments to Author:

This manuscript describes the genome sequencing and assembly of Marco Polo sheep (Ovis ammon poliii) a wild sheep species that is interesting in several regards. First, the species is adapted to living at high altitude. Second, the species is of conservation concern due to hunting, habitat loss and a limited range. Finally, there remains some uncertainty as to how often and where sheep were domesticated, and O ammon species may have contributed to the genomes of modern domestic sheep.In many ways, the manuscript is fairly routine, in the sense that the authors describe a shotgun sequencing experiment, subsequent assembly of the data and some comparative analyses of Marco Polo sheep and domesticated ovids. The work appears to have been conducted to a high standard (I lack the expertise to really judge the assembly and annotation methodologies), and it certainly looks to be thorough. It is notable that considerably more sequence data were collected (>1000 Gb) than for the assembly of the domestic sheep genome, where ~220 Gbp and ~155 Gbp of two Texel sheep was generated (Jiang et al. 2014, Science 344:1168). The N50 contig length is a little lower than that of the domestic sheep genome (~30Kb and ~40Kb respectively). Overall, the manuscript contains an impressive amount of data, it is well written, and the resource will be of interest to animal geneticists.Unsurprisingly, most of the comparisons between Marco Polo sheep and domestic sheep show very high conservation of synteny, GC content, gene number, etc. Given that these analyses are conducted independently of those done in domestic sheep, the similarity can be seen as reassurance that the analyses conducted on Marco Polo sheep genome are robust and reliable. One slight discrepancy is the average intron length (Table S13); in Marco Polo sheep this appears to be ~20% larger than is seen in domestic sheep, goats and cattle. Of course, this could be a real feature of the Marco Polo sheep genome. The authors argue that intron length appears to be conserved across the mammals they study (lines 184-185), but it might be worth trying to establish why it appears to be longer in Marco Polo Sheep.Table S20 is useful because it presents a list of genes that are putatively under positive selection. It's a bit of a shame that these are not examined or even discussed in the context of adaptation to high altitude, especially as in the Conclusion the authors comment that the genome was partially sequenced because the species is a model for studying this very question. I understand that the primary aim of the paper is to describe the resource, but nonetheless, the results presented in Table S20 warrant some discussion.On lines 142-146, the relatively low nucleotide diversity of Marco Polo sheep relative to that seen in domestic sheep is discussed. However, the sampled male was bred and reared in a zoo, and very little is said about whether the diversity is likely to be similar to that seen in wild animals. For example, if the male was inbred, because there were relatively few unrelated captive animals, then the low diversity could be unrepresentative. If the authors know that the animal is not inbred, it could be worth them stating so.

Inbreeding would be identifiable through e.g. Runs of Homozygosity.Minor typos:1) Line 405 - 'Veen' diagram should be 'Venn'.

Level of Interest

Please indicate how interesting you found the manuscript: An article of importance in its field

Quality of Written English

Please indicate the quality of language in the manuscript: Acceptable

Declaration of Competing Interests

Please complete a declaration of competing interests, considering the following questions:

- Have you in the past five years received reimbursements, fees, funding, or salary from an organisation that may in any way gain or lose financially from the publication of this manuscript, either now or in the future?
- Do you hold any stocks or shares in an organisation that may in any way gain or lose financially from the publication of this manuscript, either now or in the future?
- Do you hold or are you currently applying for any patents relating to the content of the manuscript?
- Have you received reimbursements, fees, funding, or salary from an organization that holds or has applied for patents relating to the content of the manuscript?
- Do you have any other financial competing interests?
- Do you have any non-financial competing interests in relation to this paper?

If you can answer no to all of the above, write 'I declare that I have no competing interests' below. If your reply is yes to any, please give details below.

I declare that I have no competing interests.

I agree to the open peer review policy of the journal. I understand that my name will be included on my report to the authors and, if the manuscript is accepted for publication, my named report including any attachments I upload will be posted on the website along with the authors' responses. I agree for my report to be made available under an Open Access Creative Commons CC-BY license (http://creativecommons.org/licenses/by/4.0/). I understand that any comments which I do not wish to be included in my named report can be included as confidential comments to the editors, which will not be published.

I agree to the open peer review policy of the journal
